# Single-threshold–guided adaptive cancer therapy with partial-cycle treatment: A mechanistic and reinforcement learning analysis

**DOI:** 10.1371/journal.pcbi.1014457

**Published:** 2026-06-26

**Authors:** Kexin Ma, Ningjing Wang, Zai Yang, Robert A. Cheke, Biao Tang

**Affiliations:** 1 School of Mathematics and Statistics, Xi’an Jiaotong University, Xi’an, People’s Republic of China; 2 Natural Resources Institute, University of Greenwich at Medway, Central Avenue, Chatham Maritime, Kent, United Kingdom; 3 Department of Infectious Disease Epidemiology, Imperial College London, School of Public Health, White City Campus, London, United Kingdom; 4 The Interdisciplinary Research Center for Mathematics and Life Sciences, Xi’an Jiaotong University, Xi’an, People’s Republic of China; UniversitatsSpital Zurich, SWITZERLAND

## Abstract

Adaptive cancer therapy seeks to modulate aggressive treatment to preserve drug-sensitive tumor cells that suppress resistant populations, but existing strategies often rely on frequent treatment decisions enabled by intensive surveillance, limiting clinical feasibility. Here, we propose a clinically motivated alternative that shortens the treatment window within a fixed and relatively long surveillance cycle, thereby avoiding the need for frequent monitoring. Based on this idea, we develop a mechanistic modeling framework for single-threshold-guided adaptive therapy with partial surveillance-cycle treatment (AT-PSC) and benchmark its performance using reinforcement learning. Using clinically calibrated parameters from an individual patient, simulations show that AT-PSC prolongs the time to progression (TTP) by 402 days compared with adaptive therapy using full surveillance-cycle treatment, while substantially reducing treatment exposure (dose reduced by 10.1%). Consequently, AT-PSC achieves significantly larger TTP gains than continuous therapy (1891 days) and two-threshold-guided adaptive therapy AT50 (1123 days). Simulations using data from six additional patients and sensitivity analyses further demonstrate that these benefits are robust across heterogeneous tumor growth profiles, while individual-based treatment should be considered to maximize TTP. Reinforcement learning yields comparable outcomes under the same fixed treatment window and can further extend TTP when the treatment window is adaptively adjusted. Together, these results support AT-PSC as a clinically feasible strategy to improve disease control while reducing treatment burden, and suggest that a practical regimen, such as a 14-day treatment window within a 30-day surveillance cycle, can provide sustained benefits for a broad patient population.

## Author summary

This study presents an alternative approach to adaptive cancer therapy using partial surveillance-cycle treatment (AT-PSC), aimed at reducing the need for frequent monitoring while prolonging the time to progression (TTP) and decreasing treatment exposure. The proposed framework incorporates a single-threshold-guided strategy and is validated through reinforcement learning. Simulations using clinically calibrated parameters show that AT-PSC can extend TTP by 402 days compared to full surveillance-cycle treatments, with a 10.1% reduction in drug dosage. Consequently, the approach significantly outperforms continuous therapy and two-threshold-guided adaptive therapy (AT50) in terms of TTP. Further analysis across diverse tumor growth profiles supports the robustness of the strategy, emphasizing the importance of individual-based treatment. Our results demonstrate that high-frequency monitoring is not strictly necessary to achieve the benefits of adaptive therapy. Additionally, reinforcement learning with adaptive treatment windows further enhances TTP, indicating that AT-PSC could serve as an effective, clinically feasible strategy for improving disease control while reducing treatment burden.

## Introduction

The emergence of drug resistance remains one of the major obstacles to achieving long-term remission or cure in cancer therapy [1–4]. To address this challenge, adaptive therapy was first proposed in 2009 [5], introducing an evolutionary treatment paradigm that adjusts dosing schedules in response to tumor burden.

Both clinical trials and mathematical modeling studies have demonstrated that adaptive therapy can significantly prolong the survival of patients with prostate cancer by delaying the onset of therapeutic resistance. Recently, adaptive therapy has been extended to other cancer types (e.g., breast cancer [6], melanoma [7–9], and ovarian cancer [10]), with similar benefits reported, including prolonged survival and reduced cumulative drug exposure. A central goal of adaptive therapy is to maintain an optimal balance between drug-sensitive and drug-resistant cancer cell populations. Rather than striving for complete eradication, which often accelerates the dominance of resistant clones, treatment is intermittently paused or resumed according to threshold-guided treatment rules [11–15]. This controlled cycling allows limited regrowth of sensitive cells that competitively suppress resistant ones. Consequently, the total tumor burden exhibits regulated oscillations, which remain at a relatively low level, thereby extending the time to progression (TTP) and improving patient outcomes [16–21].

With the primary goal of identifying optimal treatment schedules that maximize the time to progression (TTP) for patients, numerous mathematical models have been developed to systematically evaluate the effectiveness of adaptive therapy [22–26]. In an early and influential study published in 2017 [11], researchers conducted a clinical trial involving 11 prostate cancer patients while simultaneously developing a mathematical model to simulate a two-thresholds-guided intermittent treatment protocol. The model successfully reproduced clinical observations for several patients and provided mechanistic insights into why adaptive therapy can effectively prolong TTP, highlighting the role of competitive interactions between drug-sensitive and drug-resistant tumor cell populations. More recently, a study employing dynamic model-based reinforcement learning explored optimal treatment strategies by learning adaptive dosing policies [27]. The results suggested that a single-threshold-guided intermittent treatment [28] could serve as a practical and effective form of adaptive therapy, capable of extending TTP while reducing total drug usage compared to the two-thresholds-guided intermittent treatment in [11].

In many mathematical models, identifying an optimal treatment schedule typically requires frequent on/off decisions enabled by intensive surveillance (e.g., daily monitoring) [11,25,29,30], both of which can be clinically impractical. Recently, we proposed a general modeling framework for single-threshold-guided periodic and intermittent tumor treatment, focusing on the system’s dynamics and the existence and stability of periodic solutions [31]. In this framework, tumor burden is assessed at the start of each surveillance cycle, and treatment is initiated if it exceeds a predefined threshold. Importantly, the treatment window within each cycle can be flexibly chosen to cover only part of the surveillance period, which we refer to as adaptive therapy with partial surveillance-cycle treatment (AT-PSC). This AT-PSC design offers an alternative and clinically feasible way to de-escalate aggressive therapy, thereby prolonging TTP and reducing cumulative drug exposure without requiring frequent surveillance. Notably, while a recent study investigated a special case in which treatment occupies the entire surveillance cycle (corresponding to the AT-FSC protocol) [28], our AT-PSC framework represents a broader and more flexible generalization, potentially improving therapeutic outcomes. However, its full potential and optimal implementation remain to be systematically evaluated.

The main purpose of this study is to assess the clinical utility of the proposed AT-PSC protocol—initiating therapy by a single tumor-burden threshold while delivering treatment only during part of each (relatively long) surveillance cycle—in terms of (i) prolonging time to progression (TTP) and (ii) reducing cumulative treatment exposure. Using a mechanistic tumor–competition model with patient-specific parameters previously calibrated from clinical data, we quantify the trade-off between TTP and drug use across different choices of the threshold and treatment-window length. In parallel, we develop a mechanistic model-driven reinforcement-learning (RL) framework under the same surveillance-cycle setting to benchmark AT-PSC and to explore whether adaptive adjustment of the treatment window can yield additional benefit. Finally, we use both modeling and RL results to elucidate the underlying mechanistic rationale for AT-PSC and to derive clinically actionable guidance, including a regimen implementable in practice that can provide sustained benefit across a broad range of patients.

## Methods

### Simulation models

We simulate the tumor growth of each patient by incorporating different therapeutic strategies into the two-population Lotka-Volterra model [26], which is commonly used in adaptive therapy of tumors. The tumor cells are classified into drug-sensitive (S) and drug-resistant (R) populations. The growth dynamics of these two cell types, under treatment, can be described as follows:


$$\begin{array}{c} \left\{ \begin{array}{c} @l\frac{dS}{dt}=r_{S}S\left(\text{1}-\frac{S+\alpha R}{K}\right)(\text{1}-d_{D}D(t))-d_{S}S,\\ \frac{dR}{dt}=r_{R}R\left(\text{1}-\frac{\beta S+R}{K}\right)-d_{R}R. \end{array}\right. \end{array}$$ (1)

Here, drug-sensitive and drug-resistant cells proliferate at rates $r_{S}$ and $r_{R}$, respectively, and undergo natural death at rates $d_{S}$ and $d_{R}$. The two subpopulations share a finite environmental carrying capacity K, giving rise to resource competition. The parameters α and β represent competition coefficients quantifying competitive effects between the two cell types.

Since the primary aim of this study is to evaluate the impact of adaptive therapy strategies on prostate cancer treatment outcomes, we adopt model (1), a simplified competition-based framework, to describe tumor cell growth and drug effects. Biologically, androgen deprivation therapy (ADT) for prostate cancer acts primarily by depriving androgen-sensitive cells (S) of the hormonal signals required for cell-cycle progression, thereby inducing cell-cycle arrest (predominantly in the G0/G1 phase) rather than direct cytotoxicity [32–34]. Consequently, we model the therapeutic effect as a reduction in the intrinsic proliferation rate, represented by $r_{S}(\text{1}-d_{D}D(t))$, where $d_{D}$ represents the cytostatic efficacy of ADT and D(t) denotes the treatment intensity. This formulation aligns with established mathematical models of prostate cancer dynamics in which therapy modulates growth potential rather than increasing cell death [11,35]. Within this framework, the resistant population is assumed to be completely insensitive to ADT, and treatment-driven phenotypic conversion from sensitive to resistant cells is not explicitly included. Similar assumptions have also been used in previous studies of tumor containment, where tumors are represented by pre-existing sensitive and fully resistant subpopulations and mutations after treatment initiation are neglected [27,28]. In particular, previous work has shown that ongoing mutations after treatment initiation do not substantially affect the main containment results [36].

In all adaptive-therapy protocols considered here, a common feature is that treatment is administered intermittently in an “on/off” manner. Accordingly, we restrict D(t) to being a binary indicator function taking values in {0,1}. This choice corresponds to the clinically standard on/off regimen: during treatment-on periods, the drug is administered continuously at the standard dose(D(t)=1), whereas during treatment holidays no drug is given (D(t)=0). As a result, within our simulation framework, specifying a therapeutic strategy is equivalent to specifying the function D(t). The strategies considered in this study, together with their corresponding indicator functions, are summarized in Table 1. In the coming section, we define the different treatment strategies in detail.

**Table 1:** Treatment strategies and their treatment functions.

Strategy		Notation	Treatment Function
Continuous Therapy		CT	D(t)=1,\hspace{0.33em}∀t≥0
Intermittent Therapy		IT	$D\left(t\right)=\left\{ \begin{array}{cc} @ll@1, & t\in [n\left(T_{\text{1}}+T_{\text{2}}\right),\text{\textbackslash{}hspace\{0.17em}\}n\left(T_{\text{1}}+T_{\text{2}}\right)+T_{\text{1}}]\\ 0, & \text{otherwise} \end{array}\right.$
Two Threshold Guided Adaptive Therapy		AT50	$D\left(t\right)=\left\{ \begin{array}{cc} @ll@1, & \text{from}N\left(t\right)\geq N_{\text{0}}\text{until}N\left(t\right)<0.5N_{\text{0}}\\ 0, & \text{from}N\left(t\right)<0.5N_{\text{0}}\text{until}N\left(t\right)\geq N_{\text{0}} \end{array}\right.$
Single Threshold Guided Adaptive Therapy	Treatment in Full Surveillance Cycle	AT-FSC $\left(N_{T},T;T\right)$	$D\left(t\right)=\left\{ \begin{array}{cc} @ll@1, & N\left(nT\right)>N_{T}\text{and}t\in \left(nT,\text{\textbackslash{}hspace\{0.17em}\}\left(n+1\right)T\right)\\ 0, & \text{otherwise} \end{array}\right.$
	Treatment in Partial Surveillance Cycle	AT-PSC $\left(N_{T},T_{\text{1}};T\right)$	$D\left(t\right)=\left\{ \begin{array}{cc} @ll@1, & N\left(nT\right)\geq N_{T}\text{and}t\in (nT,\text{\textbackslash{}hspace\{0.17em}\}nT+T_{\text{1}})\\ 0, & \text{otherwise} \end{array}\right.$
Reinforcement Learning Based Adaptive Therapy		AT-RL	$D\left(t\right)=a_{t}\in \left\{0,1\right\},\text{\textbackslash{}hspace\{1em}\}a_{t}\pi_{\theta }(\cdot |s_{t})$

### Therapeutic strategies

**Continuous therapy (CT)**. Treatment is administered continuously [37–39], thus D(t)=1 for all t∈[0,TTP].

**Intermittent therapy (IT)**. There are two predefined periods: treatment window ($T_{\text{1}}$) and treatment holiday ($T_{\text{2}}$), and the treatment is alternately triggered on/off between the two periods [40–42]. Therefore, the control (treatment) time intervals can be defined as$\left\{t|t\in [n(T_{\text{1}}+T_{\text{2}}),n(T_{\text{1}}+T_{\text{2}})+T_{\text{1}}],n=\text{0},\text{1},\text{2},\ldots \right\}$.

**Two thresholds-guided adaptive therapy (AT50)**. Denote N(t)=S(t)+R(t) as the total number of tumor cells at time t, and $N_{\text{0}}$ as the initial number of tumor cells. The standard AT50 treatment protocol relies on two thresholds [11,43]: treatment is triggered (or restarted) when the tumor size exceeds $N_{\text{0}}$, and stopped when the tumor size falls below $\text{0}.\text{5}N_{\text{0}}$. Consequently, treatment remains suspended until the tumor size returns to $N_{\text{0}}$. In addition to the standard AT50, we introduce an AT50 variant for comparative analysis. In this variant, the treatment initiation threshold is defined as a variable parameter $N_{T}$ (instead of fixed $N_{\text{0}}$), while the cessation threshold remains fixed at $\text{0}.\text{5}N_{\text{0}}$; this variant is denoted as AT50$\left(N_{T},\text{0}.\text{5}N_{\text{0}}\right)$.

**Single threshold-guided adaptive therapy**. In this strategy, there is a surveillance period T of the tumor size [28,31]. At the initial time of each surveillance cycle, the decision on whether the treatment should be triggered depends on the tumor size. That is, if the tumor size exceeds a threshold $N_{T}$, the drug is used for the patients during the surveillance cycle. In addition, in line with the general framework in [31], the treatment window ($T_{\text{1}}$) can cover the full surveillance cycle ($T_{\text{1}}=T$) or just cover a partial surveillance cycle ($T_{\text{1}}<T$). Correspondingly, there are two sub-strategies as defined below:

- Adaptive therapy with full surveillance cycle treatment (AT-FSC)
- Adaptive therapy with partial surveillance cycle treatment (AT-PSC)

Correspondingly, the treatment time intervals for AT-PSC and AT-FSC are


$$\begin{array}{c} \{t|t\in (nT,nT+T_{\text{1}}),\;n=\text{0},\text{1},\text{2},\ldots \;\text{and }N(nT)>N_{T}\}, \end{array}$$

and


$$\begin{array}{c} \{t|t\in (nT,(n+\text{1})T),\;n=\text{0},\text{1},\text{2},\ldots \;\text{and }N(nT)>N_{T}\}, \end{array}$$

respectively. Note that the special adaptive therapy of AT-FSC$\left(N_{T},T;T\right)$ corresponds to the strategy studied in [28], which is therefore taken as an independent strategy for comparison.

**Fig 1:**
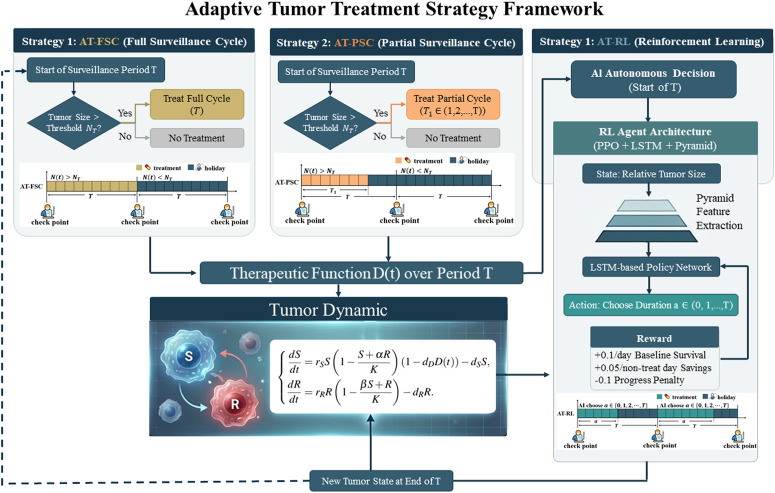
Schematic diagram of single-threshold guided adaptive therapy.

**Reinforcement learning based adaptive therapy (AT-RL)**. We develop a deep reinforcement learning (DRL) framework for adaptive tumor therapy using Proximal Policy Optimization (PPO) [44] shown in Fig 1. The environment is based on the dynamic system described in model (1). The policy/value network takes the current normalized tumor burden as input and consists of a fully connected input layer of size 32, followed by a single-layer Long Short-term Memory recurrent neural network (LSTM) with hidden size 64 and a shared multilayer perceptron with hidden dimensions 128, 64, 32, 16, and 10, before branching into separate actor and critic heads. This architecture follows the same general scalar-input + LSTM + pyramidal dense-stack design as Gallagher et al. [27], but is adapted here to our PPO-based actor–critic setting and action-space design. As in [27], the dynamic model-driven reinforcement learning approach can still be interpreted as a form of single-threshold-guided adaptive therapy, so it is not necessary to impose an explicit threshold policy in the learning process.

Similar to the single-threshold-guided adaptive therapy outlined above, we assume that the DRL agent decides whether to initiate treatment at the beginning of each surveillance cycle. In addition, we may impose a fixed treatment window within each cycle, corresponding to $T_{\text{1}}$ in the AT-PSC strategy or Tin the AT-FSC strategy. Under these settings, the reinforcement-learning-based adaptive therapy provides a framework that is consistent with the mechanistic single-threshold modeling framework and is therefore directly comparable to AT-PSC and AT-FSC. For reference, the treatment schedules for these three strategies are illustrated in Fig 1. Alternatively, as a more flexible framework for learning optimal strategies, AT-RL allows the treatment window to be adaptively adjusted in each surveillance cycle. This flexibility makes AT-RL an attractive approach for potentially further prolonging the TTP for patients.

### Parameter and simulation setting

To ensure biological plausibility and clinical relevance, we utilize a set of patient-specific parameters derived from the Phase II clinical trial of intermittent androgen deprivation therapy (IADT) conducted by Bruchovsky et al. [45]. Specifically, we directly adopt the parameter values estimated by Strobl et al. [26] (and subsequently used in [27]), which were obtained by fitting the Lotka-Volterra model (Eq. (1)) to longitudinal Prostate Specific Antigen (PSA) data. The complete parameter sets for the seven representative patients are listed in Table A in S1 Appendix. These include the initial populations of drug-sensitive cells ($S_{\text{0}}$) and drug-resistant cells ($R_{\text{0}}$), their respective growth rates ($r_{S},r_{R}$), natural death rates ($d_{S},d_{R}$), and the carrying capacity (K). Consistent with prior studies on prostate cancer competition dynamics [44], we assume that there is symmetric competition between the two subpopulations (α=β=1).

In line with the central motivation of this study, we consider a clinically realistic surveillance cycle in which tumor burden is assessed and treatment decisions are updated only at discrete follow-up visits. Following [28], we set the surveillance cycle to T=30 days, so that treatment on/off decisions are made every 30 days. Accordingly, the treatment window for AT-FSC is also 30 days. For AT-PSC, the treatment window is a fixed duration that can be chosen at any time from 0 to 30 days, providing a family of clinically feasible strategies. For intermittent therapy (IT), we set both the treatment-on period and the treatment holiday to 30 days for comparison.

Similarly, for the reinforcement learning–based adaptive therapy, the treatment window can either be fixed to a duration between 0 and 30 days in any surveillance cycle or be adaptively adjusted (within 0–30 days) across surveillance cycles. Accordingly, we focus on the following specific AT-RL strategies.

- **RL(0,30)**: Binary action a∈{0,30} days of treatment per cycle. The agent learns when to initiate a full 30-day treatment block (equivalent to data-driven AT-FSC).
- **RL(0,15)**: Action space restricted to {0,15} days. Moderate treatment durations can outperform full-cycle therapy in certain parameter regimes.
- **RL(0,19)**: The action space is {0,19} days, where $\overset{*}{\underset{\text{1}}{T}}\approx \text{1}\text{9}$ days denotes the optimal fixed treatment window for Patient 25 under the AT-PSC strategy. This value was obtained by numerically optimizing the treatment window $T_{\text{1}}$ and treatment threshold $N_{T}$ to maximize TTP.
- **RL(0,1,…,30)**: Full action space a∈{0,1,…,30} (31 discrete actions). The agent dynamically selects both whether and for how long to treat at each decision point, achieving full personalization.

As mentioned above, the simulation environment captures intra-tumoral population dynamics using the mathematical framework described in model 1. The reward function of the DRL employs a multi-objective balancing scheme that combines a baseline survival reward (0.1/day), a treatment-sparing reward (+0.05 per non-treatment day), and a progression penalty (-0.1):


$$\begin{array}{c} r_{t}\text{\textbackslash{}hspace\{0.33em}\}=\text{\textbackslash{}hspace\{0.33em}\}r_{\text{base}}\text{\textbackslash{}hspace\{0.33em}\}+\text{\textbackslash{}hspace\{0.33em}\}r_{\text{treatment}}\text{\textbackslash{}hspace\{0.33em}\}+\text{\textbackslash{}hspace\{0.33em}\}r_{\text{progress}}\text{\textbackslash{}hspace\{0.17em}\}, \end{array}$$

thus jointly targeting prolonged survival, reduced treatment burden, and delayed progression. The reward design was adapted from the reward designs used in [27] and simplified for the present tumor-dynamics setting. The reward weights were chosen heuristically rather than fitted from data, so as to encode three clinically motivated objectives on comparable scales. The policy was trained for 100,000 iterations using PPO clipping to improve training stability. As the actions are sampled from the learned policy distribution at each decision point, repeated rollouts of the same trained policy can yield different treatment trajectories and TTP values, although the environment is governed by a deterministic model.

Given the presence of a fully resistant subpopulation, complete tumor eradication is biologically infeasible in this framework. Therefore, the primary objective of our proposed policy is not cure, but containment: specifically, to decelerate tumor growth and maximize the TTP. To define this endpoint, we draw inspiration from established clinical practice. The widely used Response Evaluation Criteria in Solid Tumors (RECIST), for instance, define progression using a fixed-percentage increase in tumor burden, specifically a 20% increase relative to the size at treatment initiation [36]. Although RECIST is based on the sum of lesion diameters rather than total tumor volume, we use an analogous 20% increase in total tumor volume as a simplified model-based surrogate for clinically meaningful loss of disease control. This fixed-percentage rule is also consistent with standard practice in mathematical oncology modeling [26,36,43]. Formally, the TTP is defined as the first time that *t* satisfies:


$$\begin{array}{c} N\left(t\right)\geq 1.2\text{\textbackslash{}hspace\{0.17em}\}N_{\text{0}}. \end{array}$$

The maximum simulation horizon is set to 5000 days. If progression does not occur within this period, TTP is capped at 5000.

To quantify drug use under different treatment strategies, we report dose, defined as the cumulative abiraterone exposure delivered by a strategy relative to continuous standard of care dosing. Here, the standard of care corresponds to continuous therapy (CT), i.e., uninterrupted daily abiraterone administration until disease progression [11]. Since a constant daily dose is assumed throughout this study, cumulative drug use up to progression is proportional to the number of days on which treatment is actually given. Therefore, the dose can be computed by the following day-based ratio:


$$\begin{array}{c} Dose\%=\frac{\text{Days with treatment}}{\text{Days of TTP}}\times 100\%. \end{array}$$

### Strategy optimization

For all the threshold-guided adaptive therapies, the key control parameter is the treatment threshold $N_{T}$. There can be an optimal treatment threshold condition for maximizing TTP, which is usually denoted as $\overset{*}{\underset{T}{N}}$. Consequently, the optimized AT-FSC is denoted as AT-FSC($\overset{*}{\underset{T}{N}},T;T$), the optimized AT50 is denoted as AT50($\overset{*}{\underset{T}{N}},\text{0}.\text{5}N_{\text{0}}$), and optimized AT-PSC($\overset{*}{\underset{T}{N}},T_{\text{1}};T$). In addition to the treatment threshold, the optimization of the treatment window $T_{\text{1}}$ should also be an important aspect in prolonging the TTP, which is denoted as $\overset{*}{\underset{\text{1}}{T}}$. Therefore, there can be an optimized combination $\left(\overset{*}{\underset{T}{N}},\overset{*}{\underset{\text{1}}{T}}\right)$ that yields the maximum TTP, i.e., the optimal AT-PSC strategy denoted by AT-PSC($\overset{*}{\underset{T}{N}},\overset{*}{\underset{\text{1}}{T}};T$).

To determine the optimal patient-specific strategy, we performed a systematic grid search over the admissible treatment parameters while keeping all tumor-dynamics parameters fixed for each patient, as listed in Table A in S1 Appendix. For both AT-FSC and AT-PSC, the treatment threshold ($N_{T}$) was scanned over the interval [0.2,1.2] with an increment of 0.01. For AT-PSC, a two-dimensional grid search was further conducted over both the treatment threshold ($N_{T}$) and treatment windows $T_{\text{1}}\in \{\text{1},\text{2},\ldots ,\text{3}\text{0}\}$ days. The tumor dynamics were simulated up to 5000 days or until progression. The dynamical system was numerically integrated using SciPy’s odeint solver over each 1-day interval. To assess numerical robustness, we further repeated the analysis on refined grids and independently confirmed the main features, using a fixed-step RK4 solver (Fig A in S1 Appendix).

### Sensitivity and robustness analysis

We present the settings used to evaluate both the biological sensitivity and the robustness of the proposed AT-PSC strategy. First, we carried out a model-parameter sensitivity analysis by varying the intrinsic growth rates of the drug-sensitive and drug-resistant subpopulations, denoted by $r_{S}$ and $r_{R}$. These two parameters were selected because they directly govern the competitive growth dynamics of the sensitive and resistant cell populations in the present model and therefore provide a natural representation of inter-patient heterogeneity. Based on clinically relevant ranges reported in previous studies [26,27,46], we varied both $r_{S}$ and $r_{R}$ over the interval [0.02,0.03] using uniform grids of 11 values each. For each fixed $\left(r_{S},r_{R}\right)$ combination and treatment window $T_{\text{1}}\in \{\text{5},\text{6},\ldots ,\text{3}\text{0}\}$, we performed a global grid search over the treatment threshold $N_{T}$ on [0.2,1.2] to identify the optimal threshold that maximized TTP. The threshold-optimized AT-PSC schedule was then compared with the optimized AT-FSC schedule under the same tumor-growth parameters. In this part of the analysis, robustness was quantified by the extent to which AT-PSC maintained a positive TTP improvement over AT-FSC across a broad region of the sampled $\left(r_{S},r_{R}\right)$ space.

Second, we performed a sensitivity analysis to assess the robustness of AT-PSC with respect to clinically relevant variations in surveillance timing, such as missed appointments and holiday-induced scheduling fluctuations or delays. Taking Patient 25 as an example, we introduced stochastic appointment delays into the 30-day surveillance cycle. Specifically, the i-th surveillance interval was set to $\text{3}\text{0}+\delta_{i}$ days, where $\delta_{i}$ was independently sampled from an exponential distribution with mean μ. The mean appointment delay μ was varied from 0 to 30 days. For each nonzero value of μ, we performed 100 Monte Carlo simulations and summarized the resulting TTP by its median and interquartile range. These sensitivity analyses are model-specific and should therefore be interpreted within the assumptions of the present tumor-dynamics framework.

## Results

In this section, we first present the treatment outcomes of patients receiving the AT-PSC adaptive therapy, with the aim of evaluating its effectiveness in prolonging the TTP and reducing drug dosage compared with other therapeutic schedules. In particular, the results of the reinforcement learning–based adaptive therapy are reported in a separate subsection. Two reinforcement learning strategies are considered: one adopts a fixed treatment window within each surveillance cycle, consistent with the rule-based scheduling framework, while the other allows the treatment window to be adaptively adjusted across surveillance cycles.

### Results of AT-PSC adaptive therapy

#### *Effects of varying* $N_{T}$ *and T_1_.*

**Fig 2:**
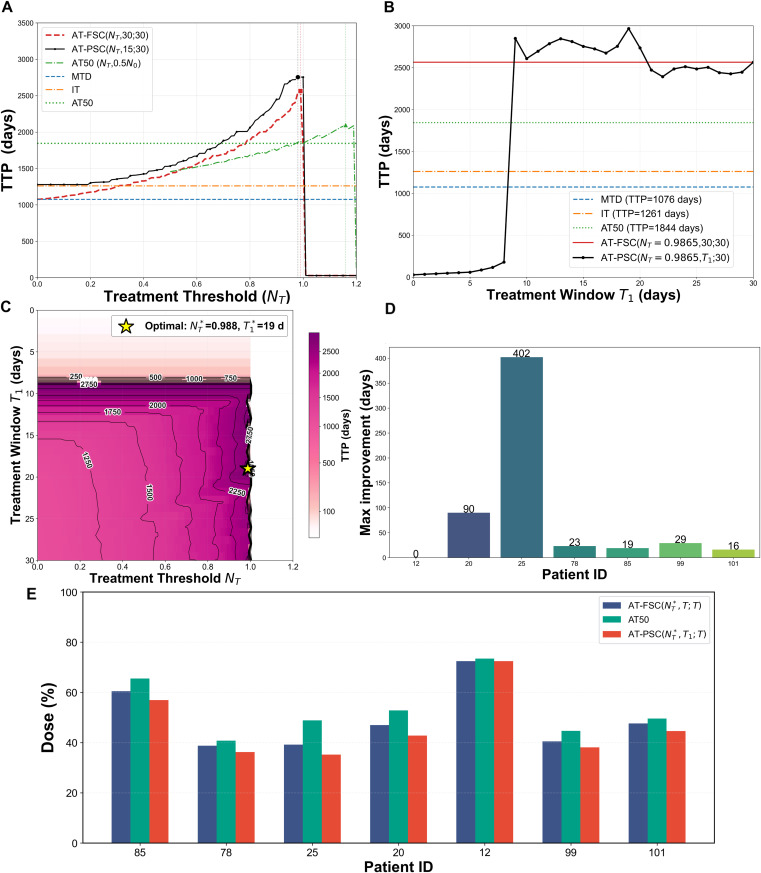
Impact of treatment threshold ($N_{T}$) and treatment window ($T_{1}$) on AT-PSC performance and comparison with other strategies. **A,** Curves of TTP as the treatment threshold $N_{T}$ increases under different strategies. Here, $T_{\text{1}}$ is fixed at 15 days for AT-PSC. **B,** Curves of TTP as the treatment window $T_{\text{1}}$ increases from 1 day to the full surveillance cycle of 30 days under different strategies. Here, $N_{T}$ is fixed at 0.9865. **C,** Heatmap of TTP under the AT-PSC strategy with respect to $N_{T}$ and $T_{\text{1}}$. The optimal strategy for maximum TTP is achieved at $\overset{*}{\underset{T}{N}}=\text{0}.\text{9}\text{8}\text{8}$ and $\overset{*}{\underset{\text{1}}{T}}=\text{1}\text{9}$ days, as marked by the yellow star. To better visualize the surface structure, the color scale is power-normalized and contour lines are overlaid. **D,** Relative improvement in TTP achieved by AT-PSC compared to AT-FSC for each patient. **E,** Dose intensity comparison across three treatment strategies for each patient.

We initially present the simulation results in Fig 2 based on the parameters related to Patient 25, with parameter values listed in the methods section. In Fig 2A, we show how the TTP varies as the treatment threshold $N_{T}$ increases for AT-PSC (marked as the black solid line), where the treatment window $T_{\text{1}}$ is fixed at 15 days within a 30-day surveillance cycle. For comparison, the TTP values of MTD, IT, and the standard AT50 strategies are plotted as horizontal reference lines, as they do not depend on the variable threshold $N_{T}$ and thus yield constant TTP outcomes. Overall, AT-PSC achieves longer TTP than the other two threshold-guided adaptive therapy strategies, AT-FSC$\left(N_{T},T;T\right)$ and AT50$\left(N_{T},\text{0}.\text{5}N_{\text{0}}\right)$, over the threshold range before the sharp decline in TTP occurs.

On the other hand, TTP increases as the treatment threshold increases before reaching the critical value ($N_{T}=\overset{*}{\underset{T}{N}}$), beyond which TTP sharply declines. Therefore, $\overset{*}{\underset{T}{N}}$ can be regarded as the optimal threshold for AT-PSC when the treatment window within each surveillance cycle is fixed, yielding AT-PSC($\overset{*}{\underset{T}{N}},T_{\text{1}};\text{3}\text{0}$). Similarly, optimal threshold values can also be identified for AT-FSC($\overset{*}{\underset{T}{N}},\text{30};\text{30}$) and AT50($\overset{*}{\underset{T}{N}}$), respectively. These optimal points are marked in Fig 2A for clarity, and the corresponding optimal treatment threshold values for each strategy are reported in Table B in S1 Appendix. A detailed comparison shows that the TTP under AT-PSC($\overset{*}{\underset{T}{N}},T_{\text{1}};\text{3}\text{0}$) is extended by 190 days (7.4%) relative to AT-FSC($\overset{*}{\underset{T}{N}},\text{3}\text{0};\text{3}\text{0}$), by 639 days (30.2%) relative to AT50($\overset{*}{\underset{T}{N}}$), and particularly by 1679 days (156%) relative to continuous treatment under MTD. This supports the conclusion that, under adaptive therapy, further reducing treatment exposure by shortening the treatment duration within each surveillance cycle can effectively extend TTP and increase the survival probability of the patients.

It should be noted that this sharp decline occurs because, once the threshold is set too high, treatment is triggered too late and becomes insufficient to control the rapid growth of sensitive cells, thereby leading to rapid progression of the total tumor burden. As a result, progression occurs before the potential benefit of adaptive therapy can be realized. This is fundamentally different from the more gradual loss of control caused by competitive release and the eventual dominance of resistant cells under excessive treatment pressure. Moreover, as shown in Fig 2A, the TTP gaps between AT-PSC, AT-FSC, and AT50 are not constant across the threshold range; rather, they are threshold-dependent and protocol-dependent, reflecting the treatment structure of each protocol.

Fig 2B illustrates how the treatment window $T_{\text{1}}$ in AT-PSC affects treatment outcomes with the treatment threshold fixed at $N_{T}=\text{0}.\text{9}\text{8}\text{6}\text{5}$, which corresponds to the optimal treatment threshold $\overset{*}{\underset{T}{N}}$ for AT-FSC under the same set of patient parameters. Since AT-PSC $\left(N_{T},T_{\text{1}};\text{30}\right)$reduces to AT-FSC $\left(N_{T},\text{30};\text{30}\right)$ when $T_{\text{1}}=\text{30}$, fixing the value of $N_{T}$ allows a direct comparison of the additional benefit gained by optimizing $T_{\text{1}}$ in AT-PSC. As shown in **Fig 2B**, once the treatment window exceeds a certain level (approximately 9 days), the TTP achieved by AT-PSC $\left(N_{T},T_{\text{1}};\text{3}\text{0}\right)$ consistently exceeds those obtained under MTD, IT, and AT50. Notably, when the treatment window $T_{\text{1}}$ is limited to a short duration (e.g., 10–20 days), the resulting TTP remains higher than that of AT-FSC. These findings additionally suggest that substantial therapeutic benefit can be achieved without continuous treatment over the entire surveillance cycle, indicating that partial-cycle treatment scheduling may effectively balance tumor control and drug exposure. In addition, there is also an optimal treatment window of AT-PSC, which is denoted by AT-PSC ($N_{T},\overset{*}{\underset{\text{1}}{T}};\text{3}\text{0}$), which has extended the TTP by 402 days (increased by 15.7%) compared to it under AT-FSC, 1123 days (increased by 60.9%) compared to it under AT50, and 1891 days (increased by 176%) compared to it under MTD.

#### *Joint optimization of* $N_{T}$ *and*$T_{1}$.

By jointly examining the results in Fig 2A and 2B, it can be seen that there exists an optimal combination of the threshold condition $N_{T}$ and the treatment window $T_{\text{1}}$ for AT-PSC. The corresponding therapeutic strategy is denoted as AT-PSC$\left(\overset{*}{\underset{T}{N}},\overset{*}{\underset{\text{1}}{T}};T\right)$. To identify this optimal combination, we constructed a contour plot of TTP with respect to $N_{T}$ and$T_{\text{1}}$, as shown in Fig 2C. For Patient 25, a full search over the two-dimensional parameter space identified the longest TTP of 2967 days at the optimal combination $\left(\overset{*}{\underset{T}{N}},\overset{*}{\underset{\text{1}}{T}}\right)=(\text{0}.\text{9}\text{8}\text{6},\text{1}\text{9}),$ which is marked by a star in Fig 2C. Such a result is consistent with the fundamental principle of adaptive therapy, which aims to judiciously modulate treatment intensity to maintain a sufficient population of drug-sensitive tumor cells capable of suppressing the growth of drug-resistant cells, rather than withdrawing treatment excessively and allowing rapid tumor expansion.

In addition, Fig 2C reveals two favorable regions of AT-PSC strategies that substantially improve TTP, suggesting two possible routes for identifying high-performing treatment schedules. The first favorable region lies near the transition boundary between high-TTP and low-TTP regions along the $N_{T}$ direction. This suggests a threshold-first strategy: one can first optimize the treatment threshold $N_{T}$, fix it near its optimal value, and then search for a suitable treatment window $T_{\text{1}}$. The second favorable region lies near the upper boundary of the high-TTP region. This suggests a window-first strategy: one can first identify a favorable treatment window $T_{\text{1}}$, fix it, and then optimize the treatment threshold $N_{T}$. These observations suggest that near-optimal AT-PSC schedules with high TTP outcomes can be identified without necessarily performing an exhaustive search over the full two-dimensional parameter space.

It is also worth noting that the white region in Fig 2C represents a treatment-failure regime of AT-PSC characterized by relatively short TTP. Fig A in S1 Appendix shows that this region persists under refined-grid analysis and independent verification using a fixed-step RK4 solver, suggesting that it is unlikely to be a numerical or search artifact. Notably, this region occurs at relatively high treatment thresholds and is therefore consistent with the sharp decline in TTP observed as $N_{T}$ increases in Fig 2A. Mechanistically, when the treatment threshold is too high, treatment initiation is delayed and the reduced treatment exposure is insufficient to suppress the drug-sensitive population. As a result, the sensitive population regrows rapidly and drives the total tumor burden to progression. This mechanism is further illustrated in Fig B in S1 Appendix using representative parameter combinations selected from the white region. Here, we emphasize that the above interpretation is restricted to the threshold-guided treatment strategies considered in this study, namely AT-PSC and AT-FSC. Thus, within the AT-PSC setting, this region is consistent with insufficient effective treatment rather than a numerical anomaly.

#### Cross-patient comparison of treatment outcomes and drug exposure.

We further evaluated the therapeutic advantages of AT-PSC$\left(\overset{*}{\underset{T}{N}},\overset{*}{\underset{\text{1}}{T}};T\right)$ in prolonging TTP while reducing drug dosage across different patients, namely the additional six prostate cancer patients described in the methods section. For each patient, we identified the optimal combination of $N_{T}$ and $T_{\text{1}}$, and summarized the corresponding TTPs under different therapeutic strategies in Table 2. To ensure a transparent comparison of the optimization effort across all strategies, we also report the optimization cost for each strategy, summarized in Table C in S1 Appendix. The results demonstrate that AT-PSC significantly extends the TTP for all seven patients compared with MTD, IT, and AT50. Moreover, with the exception of patient 12, the TTP achieved under AT-PSC is also longer than that under AT-FSC$\left(\overset{*}{\underset{T}{N}},T;T\right)$, as illustrated in Fig 2D. In particular, patient 25 exhibits the largest TTP extension, reaching 402 days. For patient 12, the optimal treatment window under AT-PSC coincides with the full surveillance cycle, such that AT-PSC reduces to AT-FSC in this case.

**Table 2:** Comparison of TTP and dose across different treatment strategies.

ID	TTP (Days)					Dose (%)		
	MTD	IT	AT50	AT-FSC^1^	AT-PSC^2^	AT-PSC	AT-FSC^3^	AT50^3^
85	682	708	751	758(0.72)	777(0.8, 26)	56.94%	60.47%(+6.20%)	65.56%(+15.13%)
78	1252	1273	1454	1468(0.84)	1491(0.84, 15)	36.26%	38.73%(+6.81%)	40.76%(+12.41%)
25	1076	1261	1844	2565(0.99)	2967(0.99, 19)	35.24%	39.20%(+11.24%)	48.83%(+38.56%)
20	1501	1550	1861	1922(0.82)	2012(0.9, 22)	42.77%	46.96%(+9.80%)	52.85%(+23.57%)
12	924	56	976	980(0.64)	980(0.64, 30)	72.48%	72.48%(+0.00%)	73.49%(+1.39%)
99	1293	1315	1498	1512(0.83)	1541(0.83, 18)	38.13%	40.52%(+6.27%)	44.70%(+17.23%)
101	1302	1343	1487	1489(0.75)	1505(0.78, 18)	44.62%	47.65%(+6.79%)	49.60%(+11.16%)

^1^Values in parentheses indicate the optimized threshold $\overset{*}{\underset{T}{N}}$.

^2^Values in parentheses indicate the optimized threshold $\overset{*}{\underset{T}{N}}$ and the optimized treatment window$\overset{*}{\underset{\text{1}}{T}}$.

^3^Values in parentheses indicate the relative percentage increase in dose compared to the AT-PSC strategy, calculated as (Strategy Dose − AT-PSC Dose)/ AT-PSC Dose ×100%.

Importantly, the observed extensions in TTP are not achieved at the cost of increased drug exposure. On the contrary, drug dosage is consistently reduced under AT-PSC for all patients, as shown in Table 2 and Fig 2E. These results indicate that the proposed AT-PSC strategy, by shortening the treatment window within each surveillance cycle, can effectively prolong TTP while simultaneously reducing overall drug exposure. This desirable “high-efficacy, low-toxicity” characteristic of AT-PSC can be intuitively concluded from Fig 2D and 2E.

**Fig 3:**
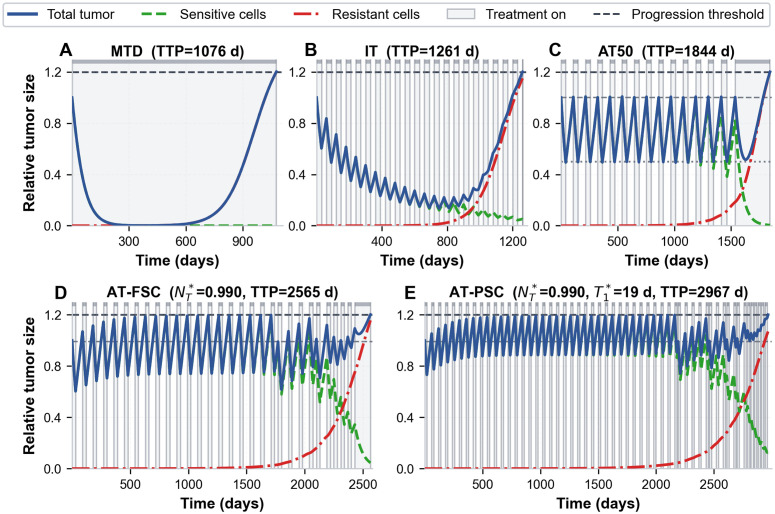
Simulation curves of relative tumor size for patient 25 under five treatment strategies.

To better understand the underlying dynamical mechanism, Fig 3 presents the evolutionary trajectories of different tumor cell populations under various therapeutic schedules for patient 25, including the outcome corresponding to AT-PSC$\left(\overset{*}{\underset{T}{N}},\overset{*}{\underset{\text{1}}{T}};T\right)$, as an illustrative mechanistic example. These trajectories provide a mechanistic explanation of how AT-PSC effectively prolongs the TTP for patient 25 by regulating the competitive dynamics between drug-sensitive and drug-resistant tumor cells. Under the AT-FSC strategy, the relative tumor size begins to drift upward during oscillations at an earlier stage, as longer treatment reduces sensitive cells excessively, weakening their competitive suppression of resistant cells and allowing the resistant population to expand. In contrast, the AT-PSC strategy maintains stable oscillations over a longer period, effectively delaying disease progression. Similarly, the evolutionary trajectories of different tumor cell populations for the remaining six patients are provided in **Figs C–H in** S1 Appendix.

These results indicate that the superiority of the AT-PSC$\left(\overset{*}{\underset{T}{N}},\overset{*}{\underset{\text{1}}{T}};T\right)$ strategy arises from its dual optimization mechanism: the threshold $\overset{*}{\underset{T}{N}}$ ensures precise timing of treatment initiation, while the parameter $\overset{*}{\underset{\text{1}}{T}}$ enables individualized adjustment of treatment duration. The synergistic effect of these two components effectively delays the expansion of the resistant cell population while maximally preserving the drug-sensitive cell population. Consequently, comparable or even superior therapeutic efficacy can be achieved with fewer treatment days, thereby substantially reducing the total drug burden on patients. This personalized treatment paradigm not only enhances efficacy but also improves treatment efficiency.

### Sensitivity and robustness analysis

In this section, we perform sensitivity and robustness analyses by varying the tumor growth parameters $r_{S}$ and $r_{R}$, which represent inter-patient heterogeneity in the proliferation rates of drug-sensitive and drug-resistant cancer cells. This analysis aims to assess the robustness and clinical effectiveness of the proposed treatment strategies in prolonging the TTP across a broad spectrum of patient-specific tumor dynamics. The results in the previous section demonstrate that the single-threshold-guided adaptive therapy proposed in [31] consistently outperforms other treatment strategies, including MTD, IT, and AT50. Building on these findings, we therefore focused on evaluating whether the AT-PSC strategy can further improve the TTP compared with the AT-FSC adaptive therapy previously studied in [28]. Unless otherwise specified, any improvement in TTP reported in this section refers to the increase in TTP achieved by AT-PSC relative to AT-FSC.

**Fig 4:**
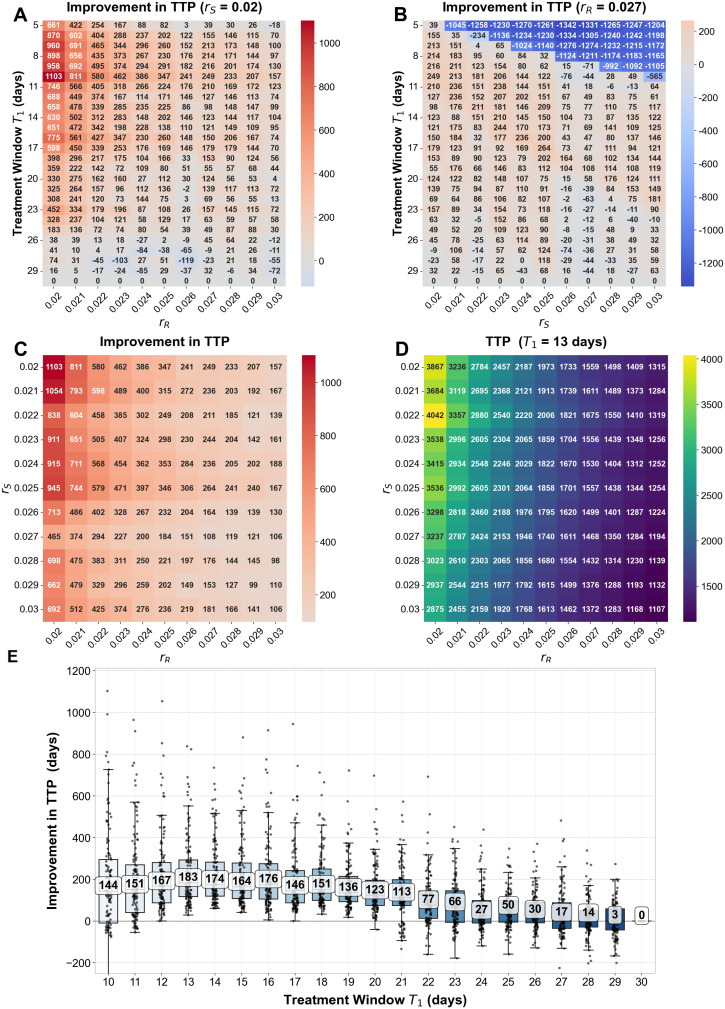
Robustness and sensitivity analysis of the AT-PSC strategy across varying tumor growth parameters. **A,** Heatmap of the improvement in TTP, varying the treatment window $T_{\text{1}}$ and the resistant cell growth rate $r_{R}$ (with $r_{S}=\text{0}.\text{0}\text{2}$ fixed). **B,** Heatmap of the improvement in TTP, varying the treatment window $T_{\text{1}}$ and the sensitive cell growth rate $r_{S}$ (with $r_{R}=\text{0}.\text{0}\text{2}\text{7}$ fixed). **C,** Heatmap of the maximum achievable improvement in TTP across the $\left(r_{S},r_{R}\right)$ parameter space, where the treatment window $\overset{*}{\underset{\text{1}}{T}}$ and treatment threshold $\overset{*}{\underset{T}{N}}$ are independently optimized for each growth rate combination. **D,** Heatmap of the TTP achieved by AT-PSC across the $\left(r_{S},r_{R}\right)$ parameter space with a fixed treatment window of $T_{\text{1}}$=13 days. **E,** Distribution of the improvement in TTP across different fixed treatment windows ($T_{\text{1}}$=10-30 days). For each value of $T_{\text{1}}$, the distribution is obtained by varying the sensitive cell growth rate $r_{S}$ and resistant cell growth rate $r_{R}$ across their respective parameter ranges.

As shown in Fig 4A and 4B, we first present two representative slices of the $(r_{S}$, $r_{R})$ parameter space by fixing one growth parameter at the fitted value for Patient 25, while varying the other growth parameter and the treatment window. These heatmaps show that, across a broad range of parameter values, there consistently exists a clinically “safe” range of treatment windows in which AT-PSC prolongs TTP relative to AT-FSC. This indicates that the therapeutic benefit of AT-PSC does not rely on fine-tuning to a single precise schedule, but can be achieved over a relatively wide window of treatment durations, enhancing its potential clinical feasibility. For each specific tumor growth profile, however, there is an optimal treatment window $\overset{*}{\underset{\text{1}}{T}}$ that maximizes TTP. Importantly, this optimal window varies substantially with $r_{S}$ and $r_{R}$, reflecting inter-patient heterogeneity in tumor growth dynamics. Moreover, we observe a clear trend that the achievable improvement in TTP diminishes as the growth rate of drug-resistant cells $r_{R}$ increases. Nevertheless, even in such cases, AT-PSC maintains a non-negligible therapeutic benefit over AT-FPC and of course the continuous MTD or AT50, supporting its potential value across diverse resistance landscapes.

In Fig 4C, we further present a heatmap illustrating the improvement in TTP achieved by the optimal AT-PSC($\overset{*}{\underset{T}{N}},\overset{*}{\underset{\text{1}}{T}};T$) relative to the optimal AT-FSC($\overset{*}{\underset{T}{N}},T;T$), as both the growth rates of drug-resistant cells $r_{R}$ and drug-sensitive cells $r_{S}$ vary from 0.02 to 0.03. Within this clinically relevant parameter range, AT-PSC robustly improves TTP by shortening the treatment window relative to AT-FSC, with the maximal extension in TTP reaching as much as 1103 days. Similar to the setting in Fig 4, additional heatmaps for different slices of the $(r_{S}$, $r_{R})$ parameter space are provided in the Supplementary Materials (Figs I–K in S1 Appendix). These supplementary results are consistent with those shown in Fig 4 and lead to the same conclusion, indicating that AT-PSC can robustly extend TTP relative to AT-FSC over a broad range of growth-rate parameters for both drug-sensitive and drug-resistant cells.

Integrating the results shown in Fig 4A–4C, a consistent pattern emerges: AT-PSC is most effective in prolonging TTP when the growth rate of drug-resistant cells is relatively low. Clinically, this corresponds to tumors in which resistant subpopulations expand slowly and remain susceptible to ecological competition from drug-sensitive cells. In contrast, when $r_{R}$ approaches or exceeds $r_{S}$, indicating aggressive resistance dynamics, the additional benefit of AT-PSC over AT-FSC is markedly reduced. This suggests that the therapeutic leverage of partial-window adaptive therapy diminishes as resistant clones gain a growth advantage.

This trend is further supported by simulations in which the treatment window is fixed at 13 days under AT-PSC, as shown in Fig 4D. As $r_{S}$ and $r_{R}$ vary over the same range, the resulting TTP spans a wide interval (from 1107 to 4042 days), reflecting substantial inter-patient variability. Notably, the longest TTP (4042 days) is achieved when both $r_{S}$ and $r_{R}$ are at their lower values ($r_{S}=\text{0}.\text{0}\text{2}\text{2},r_{R}=\text{0}.\text{0}\text{2}$), reinforcing the clinical insight that tumors with slower growth and weaker resistance dynamics are most amenable to benefit from adaptive strategies that reduce treatment intensity.

Although the above results suggest that individualized therapeutic strategies should be designed to maximize the TTP for each patient, from a clinical perspective, it is also desirable to identify a unified treatment schedule that can deliver sustained benefits across a broad range of patients. That is, instead of relying solely on patient-specific optimization, we aim to identify a fixed treatment window of AT-PSC that can robustly improve the TTP for most patients. To this end, in **Fig 4E**, we plot the distribution of the improvement in TTP of AT-PSC (resulting from variations in $r_{S}$ and $r_{R}$) across different treatment windows, relative to AT-FSC, showing that the median improvement in TTP exhibits a non-monotonic dependence on the treatment window.

In particular, for $T_{\text{1}}$=10–20 days the median TTP improvement consistently exceeds 120 days (approximately 123–183 days), peaking at 183 days when $T_{\text{1}}$=13 days. Meanwhile, the interquartile ranges of the box plots are comparable across these windows, indicating limited sensitivity to moderate changes in the treatment window. On the other hand, Fig 4E shows that treatment windows in the range of approximately 12–16 days can robustly improve TTP relative to AT-FSC. In view of this robustness, together with the practical convenience of a two-week-on/two-week-off schedule within a 30-day surveillance cycle, we consider a constant AT-PSC schedule with a fixed 14-day treatment window (i.e., a “14-30” schedule) as a feasible and clinically interpretable candidate schedule within the present modeling framework.

To further examine whether this conclusion depends on treating all sampled parameter combinations equally, we repeated the analysis by assigning larger weights to parameter combinations closer to the fitted growth rates of the seven patients during sampling, as shown in Fig L in S1 Appendix. Under this setting, both the median and mean improvements in TTP were also maximized at $T_{\text{1}}=\text{1}\text{3}$ days, consistent with the result shown in Fig 4E. Moreover, the results in Fig L in S1 Appendix further suggest that a “14-30” schedule may serve as a feasible and clinically interpretable candidate schedule. Therefore, the main conclusion drawn from Fig 4E is not dependent on uniform parameter sampling.

Beyond the above sensitivity analysis, we further examined the robustness of AT-PSC with respect to surveillance intervals and appointment delays in monitoring. First, we re-optimized the AT-PSC schedule under different surveillance intervals for each patient-specific parameter set, as shown in Fig M in S1 Appendix. Fig M in S1 Appendix indicates that TTP can be very sensitive to the surveillance interval depending on patient-specific parameters. For example, more frequent surveillance may yield a longer TTP for Patient 25. However, for many of the other patients, TTP changed only slightly as the surveillance interval varied from 1 to 60 days. For comparison, we further evaluated the optimized AT-FSC and AT-PSC strategies across the same set of surveillance intervals, and the corresponding TTP heatmaps are shown in Fig N in S1 Appendix. The results show that, across different surveillance intervals, AT-PSC generally yields longer TTP than AT-FSC for most patients.

We then examined the sensitivity of treatment outcomes to stochastic appointment delays under a 30-day surveillance cycle, as shown in Fig O in S1 Appendix. The results indicate that imperfect adherence to the planned monitoring schedule, represented here by appointment delays, generally reduces TTP, with the maximal TTP usually achieved in the absence of delays. In particular, when the treatment threshold was optimized under the no-delay setting, the resulting strategy was highly sensitive to appointment delays, leading to a rapid decrease in TTP as the mean delay increased. In contrast, lowering the treatment threshold improved robustness to moderate delays. When the mean delay was less than 7 days, TTP could remain close to that achieved by the no-delay AT-PSC strategy, although this robustness came at the cost of reducing the maximal TTP gain under ideal no-delay conditions.

### Results of reinforcement learning based adaptive therapy

**Fig 5:**
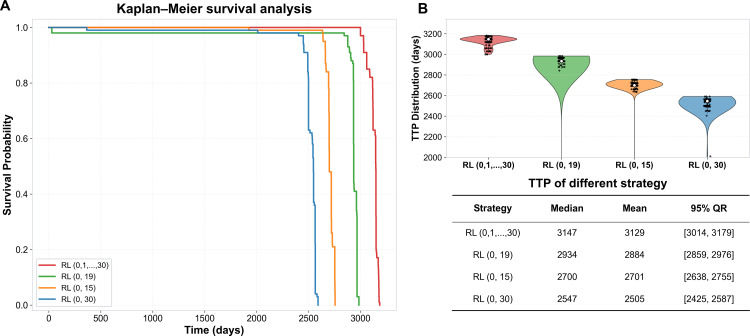
Impact of action space design on RL-based adaptive therapy performance under the parameter set of Patient 25. **A,** Kaplan–Meier curves showing progression-free survival evaluated on a cohort of 100 stochastic simulations for four RL strategies with different action spaces: full action space (0-30 days) and binary action spaces (0/19, 0/15, 0/30 treatment days per cycle). **B,** Violin plots depicting the TTP distribution for each strategy across the same 100 simulations with the underlying individual data points overlaid. The accompanying table summarizes key statistics including median, mean, and 95% quantile range.

We first report the results of the reinforcement learning (RL)-based adaptive therapy with a fixed treatment window within each surveillance cycle, aiming to learn an effective adaptive treatment strategy for patient 25. Consistent with the setting of our mechanistic modeling framework, we consider three RL schedules: full-cycle treatment RL(0,30), and partial-cycle treatment with a fixed treatment window of 15 days RL(0,15) and 19 days RL(0,19), where 19 days corresponds to the patient-specific optimal treatment window $\overset{*}{\underset{\text{1}}{T}}$ identified for Patient 25 under the AT-PSC framework. The results are shown in Fig 5.

The survival analysis in Fig 5A indicates that RL(0,30) tends to be less effective in prolonging survival (or TTP) than the partial-cycle treatment strategies. This finding is consistent with the results obtained under the rule-based protocols and further supports the clinical relevance of reducing the treatment window as a feasible way to de-escalate aggressive therapy while improving disease control. More specifically, RL(0,19) appears to outperform RL(0,15), yielding an additional improvement of approximately 234 days in TTP. Notably, the 19-day treatment window is also identified as optimal for Patient 25 under the AT-PSC framework, and the median TTP obtained by RL under this fixed 19-day constraint is 2934 days, which is comparable to the 2967 days achieved by the optimized AT-PSC strategy. Taken together, these results provide further evidence that our modeling framework can offer an effective mechanistic design of adaptive therapy schedules, yielding sustained improvements in TTP while maintaining clinical feasibility.

In Fig 5, we further present the results of the RL-based adaptive therapy in which the treatment window is adaptively adjusted from 1 day to 30 days, denoted as RL(0,1,...,30). The results show that the median TTP can be further extended by 213 days compared with RL(0,19). This advantage is also reflected in the violin plots, where RL(0,1,…,30) exhibits a higher median TTP and a narrower interquartile range, indicating more consistent performance across repeated rollouts. Note that the Kaplan–Meier curves in Fig 5A show a relatively steep decline in survival probability across all strategies. Fig 5 was generated from 100 stochastic simulations for a cohort of virtual patients sharing the same parameter set (Patient 25). In these simulations, the only source of variation is the stochastic action selection from the trained RL policy at each decision point, while the underlying tumor dynamics remain deterministic. The steep drop therefore indicates that progression times are relatively concentrated, with many virtual patients reaching disease progression within a narrow time window, rather than being broadly dispersed. This suggests that, under the learned RL strategy and the fixed cycle-based decision interval, the simulated treatment trajectories are relatively consistent across repeated rollouts. This pattern is consistent with the observation in [27].

**Fig 6:**
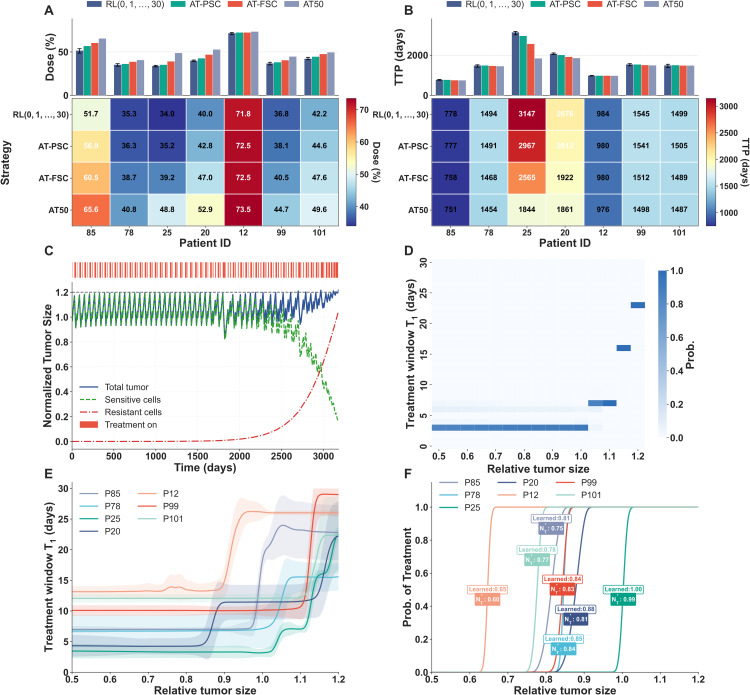
Performance and learned treatment-window policies of RL compared with rule-based treatment strategies. **A,** Time to progression (TTP) and **B,** dose under RL(0,1,...,30), AT-PSC, AT-FSC, and AT50. For RL(0,1,...,30), the plotted values are the medians over repeated stochastic simulations, and error bars indicate the 95% quantile range. **C,** Tumor trajectory for Patient 25 under the adaptive RL(0,1,…,30) strategy, shown for the rollout achieving the maximal TTP, together with the corresponding within-cycle treatment schedule. **D,** Action probability heatmap for Patient 25 under RL(0,1,…,30), illustrating the probabilities of selecting treatment windows $T_{\text{1}}\in \{\text{0},\text{1},\ldots ,\text{3}\text{0}\}$ at different levels of relative tumor size. **E,** Expected treatment window length under RL(0,1,…,30) across relative tumor sizes for the seven patients. Solid lines represent the smoothed expected value of the treatment window, and shaded bands show the corresponding smoothed dispersion (mean ± standard deviation) computed from the action-probability distribution. **F,** Treatment selection probability curves under the binary action RL policy (treat for a fixed window $\overset{*}{\underset{\text{1}}{T}}$ vs. no treatment) for the seven patients, plotted against relative tumor size. Here, $\overset{*}{\underset{\text{1}}{T}}$ denotes the patient-specific optimized treatment window reported in Table 2. Curves are smoothed for visual clarity. The implied switching threshold learned by the agent is highlighted in the white boxed region, and the reference threshold $\overset{*}{\underset{T}{N}}$ is indicated for comparison.

In addition, we evaluated the performance of RL(0,1,…,30) against other strategies for the seven prostate cancer patients, with results summarized in Fig 6 (exact values are provided in Table D in S1 Appendix). The four strategies exhibit a clear performance ordering in terms of TTP: RL(0,1,…,30) performs best, followed by AT-PSC, AT-FSC, and AT50. The differences are visually apparent in Fig 6A, and RL(0,1,…,30) achieves longer TTP for all patients, with maximum gains of 180 days relative to AT-PSC, 582 days relative to AT-FSC, and 1303 days relative to AT50. RL may generate different treatment trajectories across independent runs; therefore, we report the median TTP for comparison. Importantly, the treatment schedule for each trajectory can be explicitly recorded. The treatment schedule corresponding to the highest TTP can then be substituted into the deterministic model to reproduce the same trajectory and outcome, as the RL training environment is constructed from the same deterministic model. This indicates that the RL-learned schedules, particularly the schedule achieving the longest TTP, are feasible within the proposed modeling framework. Moreover, as shown in Fig 6B, RL(0,1,…,30) not only prolongs TTP but also reduces cumulative drug use, with maximum dose reductions of 9.26% relative to AT-PSC, 14.81% relative to AT-FSC, and 30.37% relative to AT50.

Further, we explore the mechanism behind how the RL(0,1,…,30) adaptive therapy can achieve superior tumor control, compared with the treatment strategy with a fixed treatment window. To this end, we first show, for Patient 25, the evolution of the two tumor cell populations under RL(0,1,…,30) for the simulation run that achieves the maximal TTP (Fig 6C), together with the corresponding action selection probabilities across different tumor size states (Fig 6D). Note that the strategy learned from RL(0,1,…,30) extended the TTP by approximately 8.2% (3210 days vs. 2967 days) compared with the AT-PSC ($\overset{*}{\underset{T}{N}},\overset{*}{\underset{\text{1}}{T}};T$) strategy, and prolonged it by 234 days compared with RL(0,19).

From Fig 6D, when the relative tumor size is below 1, the action probabilities under RL(0,1,…,30) concentrate on very short treatment windows ($T_{\text{1}}=\text{3}$days). This indicates that in the early, low-burden stage, RL(0,1,…,30) tends to use short window dosing to reduce treatment exposure while preserving the competitive advantage of drug-sensitive cells. As the tumor size increases to around 1.05, the preference for short windows rapidly diminishes and shifts to a moderate window ($T_{\text{1}}=\text{7}$days), suggesting that stronger within-cycle intervention is required as the system enters a higher-risk state. When the tumor burden further rises into the high-risk range (approximately 1.1-1.2), RL(0,1,…,30) increasingly favors longer windows ($T_{\text{1}}=\text{1}\text{6}$days), and as the tumor approaches the progression boundary it further shifts toward $T_{\text{1}}=\text{2}\text{3}$ days. Analogous policy heatmaps for all seven patients are provided in Fig P in S1 Appendix. This pattern reflects a more aggressive intensification of treatment in high-risk states to suppress tumor growth and avert rapid progression. Notably, this early de-escalation in treatment intensity is consistent with a delayed expansion of the drug-resistant subpopulation, as can be seen by comparing Fig 6C with 3E.

This phenomenon suggests that the RL strategy may imply a multi-threshold treatment approach: different treatment windows are set based on the tumor burden at various stages. When the tumor size exceeds a certain threshold, the treatment window increases to address higher-risk states. In the early stages, the RL strategy tends to use short treatment windows to avoid overtreatment; as the tumor progresses, the window gradually extends, allowing for more precise treatment control. This mechanistic interpretation is further supported by Fig 6E, where the RL agent adaptively increases the treatment window from a relatively short duration in the initial phase as the tumor size grows for the seven patients.

In contrast, under the fixed treatment window constraint, the strategy learned by RL primarily manifests as a single threshold decision rule, as illustrated in Fig 6F. Specifically, when facing the binary choice of $\left\{\text{0},\overset{*}{\underset{\text{1}}{T}}\right\}$ at each surveillance cycle, the agent learns to make decisions based on relative tumor size: it tends to pause treatment when the tumor size is below a specific level, and triggers a treatment once the threshold is exceeded. We define the relative tumor size corresponding to a treatment probability exceeding 0.5 as the agent’s learned “treatment threshold.” Notably, these learned thresholds (labeled as “Learned”) align closely with the theoretical optimal threshold $\overset{*}{\underset{T}{N}}$ derived from the single-threshold AT-PSC framework. This suggests that, under identical fixed treatment window constraints, AT-PSC has achieved a near-optimal switching strategy. This consistency explains why the fixed window RL and the optimized AT-PSC scheme yield comparable TTP outcomes. Specifically, the RL approach with the fixed treatment window $\overset{*}{\underset{\text{1}}{T}}=\text{1}\text{9}$ days achieves a median TTP of 2934 days (95% quantile range: [2859, 2976]; Fig 5B), which is close to the 2967 days for the optimized AT-PSC scheme (Fig 3E). To provide a complete comparison of the RL formulations, we further summarize their action-space definitions, network sizes, and benchmark runtimes in Table E in S1 Appendix.

## Discussion

In this study, motivated by the clinical challenge that frequent surveillance is often required to support precise treatment decisions, we propose an adaptive therapy strategy in which treatment is delivered only during part of each surveillance cycle while the surveillance interval remains relatively long. Building on this idea, we develop a mechanistic modeling framework for adaptive therapy with partial surveillance-cycle treatment, termed AT-PSC. In parallel, we developed a reinforcement learning-based adaptive therapy framework that follows the same principle-administering treatment only during a specified portion of each surveillance cycle. We then systematically evaluated the clinical utility of this partial surveillance-cycle treatment strategy in terms of prolonging time to progression (TTP) and reducing cumulative drug exposure, and compared its performance against several widely used alternatives, including single-threshold–guided adaptive therapy with full-cycle treatment, AT50, periodic intermittent therapy, and continuous treatment (MTD).

By fixing the surveillance cycle at 30 days, we simulated tumor burden and drug exposure across a broad range of patients, using both clinical data from seven prostate cancer patients (with parameters calibrated from clinical data) and a broader patient population through sensitivity analysis. The results indicate that, compared to AT-FSC, AT50, and MTD, AT-PSC consistently prolongs the time to progression (TTP) while reducing cumulative drug exposure. In particular, for the seven patients, AT-PSC achieved a maximum TTP gain of 402 days relative to AT-FSC (full-cycle treatment) and a maximum reduction of 11.24% in drug dosage (see Fig 2 and Table 2). Consequently, AT-PSC achieved a significant improvement in TTP compared with AT50, IT, and MTD, as the AT-FSC has been proven to be effective in prolonging the TTP compared with other strategies.

The conclusion is supported by the reinforcement learning (RL) experiments for adaptive therapy. Under a fixed treatment-window constraint, the treatment thresholds learned by RL are highly consistent with the theoretical optimal threshold $\overset{*}{\underset{T}{N}}$ in the AT-PSC framework. This agreement suggests that, under this constraint, the AT-PSC partial window adaptive therapy strategy already achieves near-optimal switching performance, while RL provides an independent validation. It should be mentioned that the RL agent does not converge to a single fixed rule; instead, it exhibits dynamically evolving adjustment behavior over the course of therapy in response to patient-specific tumor dynamics. By analyzing the action-probability distributions output by the policy network, we can characterize the agent’s distinct decision patterns across patients. Moreover, by leveraging long-term return evaluation through the value function, the RL strategy can adjust treatment intensity more precisely under continued cycle-based surveillance of tumor status.

In addition to the fixed treatment window for each surveillance cycle, for the strategy of adaptive shifting of treatment window in RL, i.e., RL(0,1,…,30), the agent is allowed to choose the treatment window dynamically within each surveillance cycle. Compared with the optimized AT-PSC strategy that relies on fixed thresholds and a fixed window, the RL with adaptive adjusted treatment window achieves longer TTP, with an additional improvement of up to 234 days (see Fig 6C) and further reduces the dose in most patients (maximum reduction 9.26%, see Table D in S1 Appendix). Collectively, these results indicate that, at later disease stages or under more complex tumor dynamics, more flexible dynamic window adjustment may yield additional benefit, while also offering clearer decision support for clinical practice.

It should be emphasized that this observation may provide useful insight into the design of new treatment schedules for further prolonging TTP. Specifically, it suggests the possibility of a multi-threshold-guided adaptive therapy strategy, in which the treatment window is allowed to vary across adjacent threshold intervals. As indicated by Fig 6E, the treatment window between two adjacent thresholds may need to increase as the threshold level increases. In practice, at each surveillance visit, the current tumor burden would be mapped to a predefined threshold interval, and the corresponding treatment window would then be assigned. Under this setting, however, the optimization problem becomes more complex, as it would involve not only the selection of multiple thresholds but also the combination of treatment windows assigned to different threshold intervals. Moreover, the noticeable variation across patients suggests that the threshold set and the associated treatment windows would probably need to be individualized, for example by calibrating a mechanistic model to each patient’s early treatment-cycle data, or, at the cohort level, by identifying representative patient subgroups and deriving subgroup-specific protocols. Therefore, a dedicated mechanistic modeling framework for multi-threshold-guided adaptive tumor therapy would be required to systematically evaluate the effectiveness of this strategy. We believe that this represents an interesting direction for future research and may offer a promising opportunity to further improve adaptive therapy designs aimed at prolonging TTP.

To provide the mechanical understanding, we present the evolution trajectory of the tumor cells under different therapeutic schedules, showing that the AT-PSC jointly controls the treatment threshold $N_{T}$ and the treatment window length $T_{\text{1}}$. This transforms the rigid periodic paradigm of “once treatment begins, it covers the entire cycle” into a controllable plan where treatment is delivered only within a prescribed window. Consequently, AT-PSC can effectively balance between “tumor suppression” and “maintenance of competitive pressure.” Delivering therapy only within the treatment window can effectively reduce tumor burden early in each cycle, while the subsequent drug holiday avoids sustained over-elimination of drug-sensitive cells, thereby preserving their ecological competition against resistant cells and slowing the rise of the resistant fraction. Consequently, under fixed surveillance, the windowed dosing design of AT-PSC is more effective at preserving the sensitive population and restraining resistant expansion, leading to more durable clinical benefit.

The results indicate that the improvement in TTP is highly dependent on the parameter settings, making it sensitive to individual patient characteristics. Therefore, individualized strategies should be considered when aiming to maximize the TTP for each patient. However, we also propose a unified therapeutic schedule for AT-PSC aimed at prolonging TTP and reducing drug exposure across a broad range of patients. Specifically, we demonstrated that a stable positive benefit is achieved for treatment windows of $T_{\text{1}}$ = 10–20 days within a 30-day surveillance cycle (with median improvements exceeding 120 days; see Fig 4E). Moreover, an AT-PSC strategy with a fixed treatment window of 14 days within a 30-day surveillance cycle may serve as an effective and robust approach to improving TTP across a wide patient population.

There are still several limitations in this study. First, the model neglects spatial structure and microenvironmental heterogeneity, both of which can alter intra-tumoral competition and thereby influence the efficacy of adaptive therapy [47–50]. Previous studies have characterized tumor spatial heterogeneity using spatially resolved approaches, including microdissection-based genotyping of distinct tumor regions and digital pathology-based mapping of tumor microenvironmental components [47–50]. These studies suggest that spatial organization can create locally distinct tumor subpopulations and region-specific selective pressures, such as hypoxia and nutrient limitation, thereby reshaping intratumoral competition, treatment pressure, and therapeutic response. As a result, the quantitative benefit of AT-PSC may differ in more spatially structured tumors. Future studies could extend AT-PSC to spatially heterogeneous tumor models to evaluate how spatial heterogeneity affects its therapeutic benefit and optimal scheduling. Second, the current model assumes that the resistant population is fully insensitive to ADT and does not explicitly include treatment-driven phenotypic transitions from sensitive to resistant states [51]. Experimental and mathematical studies have suggested that resistance under androgen-deprivation therapy may involve multiple mechanisms, including treatment-associated adaptation or phenotypic switching, and that prolonged hormone-deprived conditions can promote neuroendocrine transdifferentiation and support progression toward hormone-refractory disease [51–53]. Therefore, our conclusions should be interpreted as applying to a simplified competition-based scheduling framework, rather than to the full spectrum of prostate cancer resistance evolution. Extending the present model to include partial drug sensitivity of the resistant compartment, therapy-driven switching, or additional resistant phenotypes would be an important direction for future work. Third, we do not explicitly model pharmacokinetics and pharmacodynamics (PK/PD), assuming a constant drug dose and a constant effect during treatment, which may overestimate the instantaneous cytotoxic impact in real clinical settings. Future work incorporating spatially explicit models and PK/PD processes would be valuable and could provide further insights into the robustness and translational potential of unified treatment schedules, such as the “14-30” schedule, using larger patient cohorts.

Despite the above limitations, our results indicate that AT-PSC is a strategy with high potential for clinical translation. This approach effectively prolongs TTP while significantly reducing cumulative drug exposure, which not only helps to mitigate treatment-related toxicities but also alleviates the economic burden on patients. More importantly, the “fixed surveillance cycle combined with dosing within the treatment window” paradigm adopted by AT-PSC may provide a clinically interpretable framework for adaptive-therapy design in chronic malignancies such as prostate cancer that require long-term management. However, further model refinement, biological validation, and clinical evaluation will be required before specific schedules such as the 14–30 strategy can be considered for practical implementation.

### Ethics statement

No ethics approval was required for this study, as all data used were obtained from publications for which ethical approval had been obtained.

## Supporting information

S1 Appendix Supplementary analyses, figures, and tables. This supporting document contains all supplementary notes, figures, and tables cited in the main text, including validation of the white region in the AT-PSC parameter scan; tumor-dynamics plots for the remaining six patients; parameter-space analyses of AT-PSC performance across tumor growth profiles and treatment-window settings; empirically weighted analysis of treatment-window selection; robustness analyses of AT-PSC to surveillance intervals and appointment delays; policy heatmaps across patients; virtual patient parameters; protocol-specific optimal thresholds for the threshold-guided adaptive therapy strategies; optimization cost of rule-based treatment strategies; outcomes under adaptive RL(0,1,…,30) compared with other treatment strategies; and computational cost of the RL-based strategies.
(PDF)
