# Supplementary material for: Single-threshold–guided adaptive cancer therapy with partial-cycle treatment: A mechanistic and reinforcement learning analysis
Source: PLoS Comput Biol. 2026 Jun 26;22(6):e1014457. doi: 10.1371/journal.pcbi.1014457 (PMC13336478; doi:10.1371/journal.pcbi.1014457)
Supplement: S1 Appendix — This supporting document contains all supplementary notes, figures, and tables cited in the main text, including validation of the white region in the AT-PSC parameter scan; tumor-dynamics plots for the remaining six patients; parameter-space analyses of AT-PSC performance across tumor growth profiles and treatment-window settings; empirically weighted analysis of treatment-window selection; robustness analyses of AT-PSC to surveillance intervals and appointment delays; policy heatmaps across patients; virtual patient parameters; protocol-specific optimal thresholds for the threshold-guided adaptive therapy strategies; optimization cost of rule-based treatment strategies; outcomes under adaptive RL(0,1,…,30) compared with other treatment strategies; and computational cost of the RL-based strategies. (PDF) [file pcbi.1014457.s001.pdf]

S1 Appendix

Single-Threshold–Guided Adaptive Cancer Therapy with Partial-Cycle Treatment: A Mechanistic and Reinforcement Learning Analysis

Kexin Ma[1], Ningjing Wang[1], Zai Yang[1], Robert A Cheke[2,3,*], Biao Tang[1,4,**]

1. School of Mathematics and Statistics, Xi'an Jiaotong University, Xi'an, People's Republic of China

2. Natural Resources Institute, University of Greenwich at Medway, Central Avenue, Chatham Maritime, Kent, United Kingdom

3. Department of Infectious Disease Epidemiology, Imperial College London, School of Public Health, White City Campus, London, United Kingdom

4. The Interdisciplinary Research Center for Mathematics and Life Sciences, Xi'an Jiaotong University, Xi'an, People's Republic of China

[*]Correspondence: R.A.Cheke@greenwich.ac.uk (Robert A Cheke)
[**]Correspondence: biaotang@xjtu.edu.cn (Biao Tang)

Supplementary Note 1. Validation of the white region in the AT-PSC parameter scan

To examine whether the white region observed in Fig. 2C is a numerical or search artifact, we performed additional validation analyses within the AT-PSC setting. First, we repeated the parameter scan using a locally refined grid around the white region. The low-TTP structure persisted under the finer discretization. Second, we independently repeated the same local scan using a fixed-step RK4 solver, which reproduced essentially the same low-TTP pattern. Third, we quantified the effective treatment exposure and the first treatment start day over the same parameter region. We found that the white region coincides with markedly reduced on-treatment exposure and substantially delayed treatment initiation. Finally, representative TTP profiles across the white region were highly consistent between the odeint-based and RK4-based computations. To further illustrate the underlying mechanism, Fig B shows representative tumor-dynamics trajectories for several parameter combinations within the white region. These examples indicate that, when the threshold N_T is relatively high and the treatment window T_1 is short, treatment is initiated too late and maintained for too short a duration to effectively suppress tumor growth. Under this condition, the sensitive population is not sufficiently controlled, and the total tumor burden increases steadily, reaching progression within a relatively short time. Taken together, these results indicate that the white region is not caused by coarse grid resolution or solver-specific numerical

artifacts. Rather, within the AT-PSC setting, it is consistent with insufficient effective treatment, characterized by too little treatment exposure and overly delayed treatment initiation.

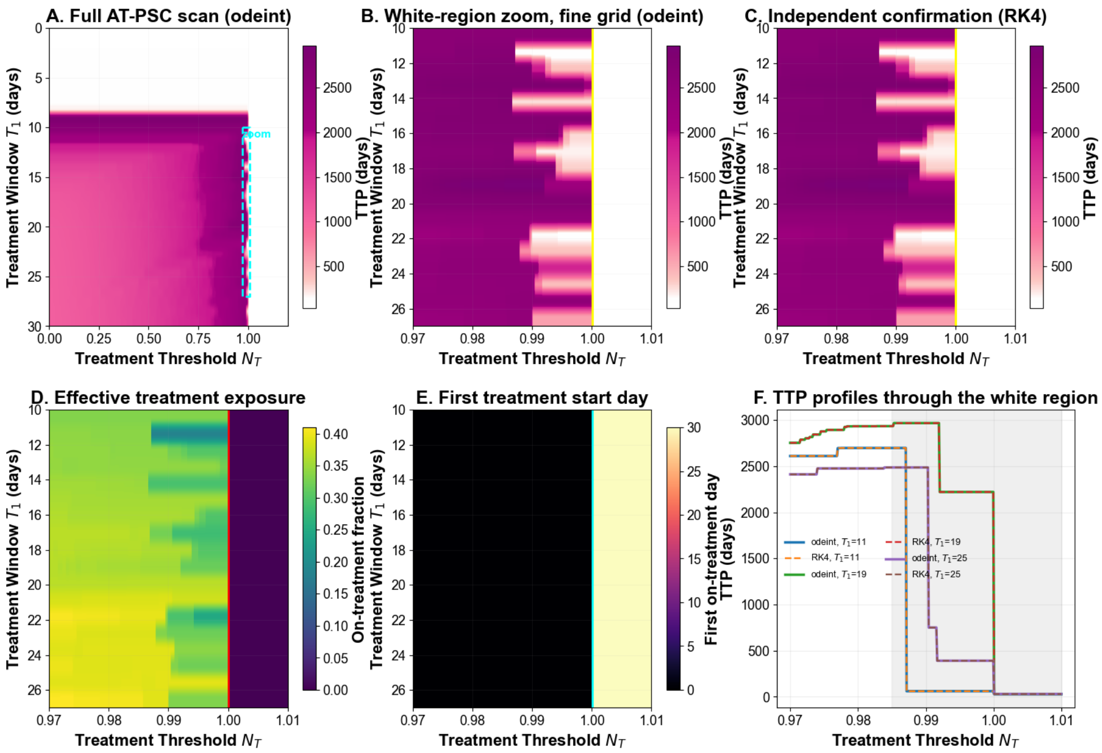

Fig A. Validation of the white region in the AT-PSC parameter scan. (A) Full AT-PSC scan computed using odeint, with the white-region area highlighted. (B) Local zoom-in of the white region using a refined grid and odeint. (C) Independent confirmation of the same local region using a fixed-step RK4 solver. (D) Effective treatment exposure, measured by the fraction of time under treatment, over the same local parameter region. (E) First treatment start day over the same local parameter region. (F) Representative TTP profiles across the white region for selected values of T_1, comparing odeint and RK4.

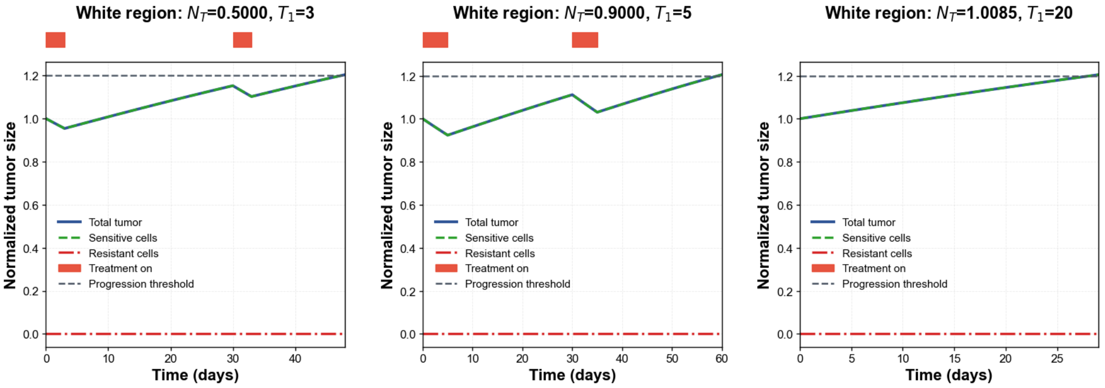

Fig B. Representative tumor-dynamics trajectories for parameter combinations within the white region of the AT-PSC parameter scan.

Supplementary Note 2. Tumor-dynamics plots for the remaining six patients

To complement the representative tumor-dynamics results shown in the main text, we provide the corresponding plots for the remaining six patients in Figs C–H. For each patient, the tumor burden trajectories under MTD, IT, AT50, AT-FSC, and AT-PSC are shown together with the temporal evolution of the sensitive and resistant subpopulations. These figures illustrate that the qualitative advantage of AT-PSC over the comparator strategies is consistently associated with improved preservation of the sensitive population and slower expansion of the resistant population, although the magnitude of benefit varies across patients.

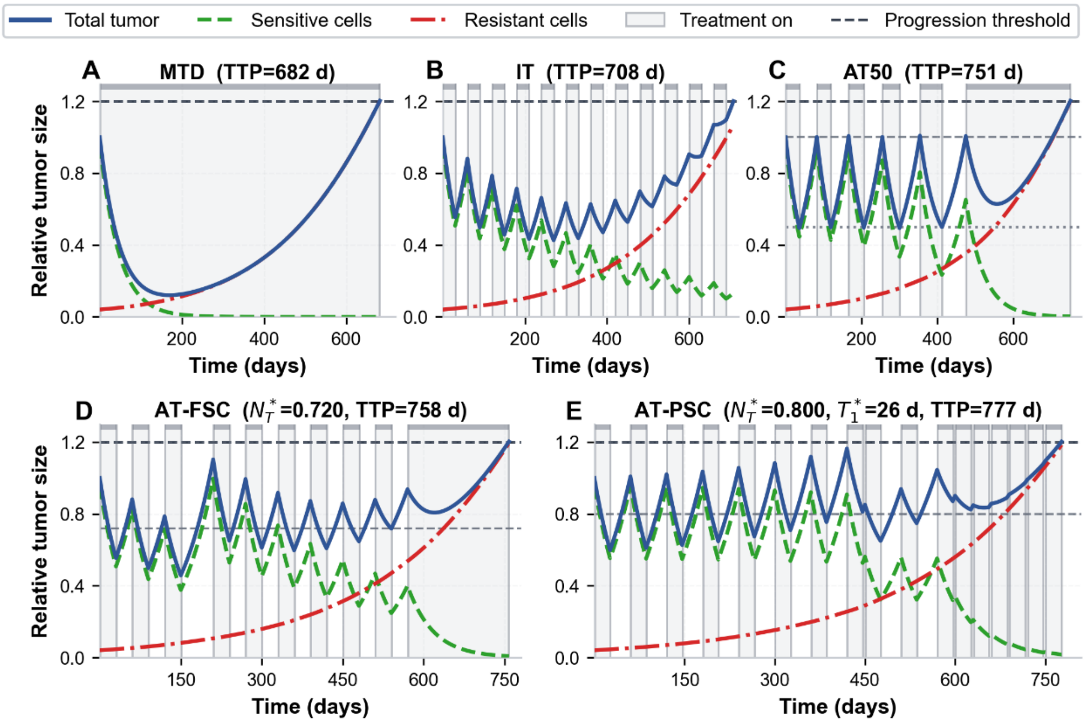

Fig C. Tumor-dynamics plots for Patient 85 under different treatment strategies.

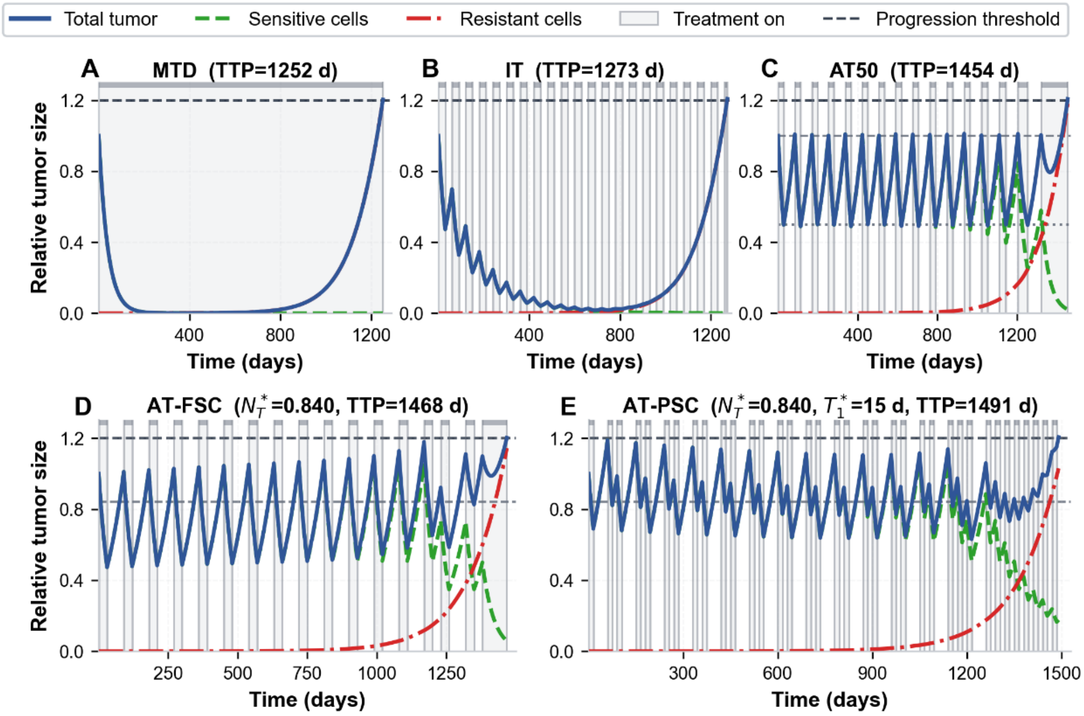

Fig D. Tumor-dynamics plots for Patient 78 under different treatment strategies.

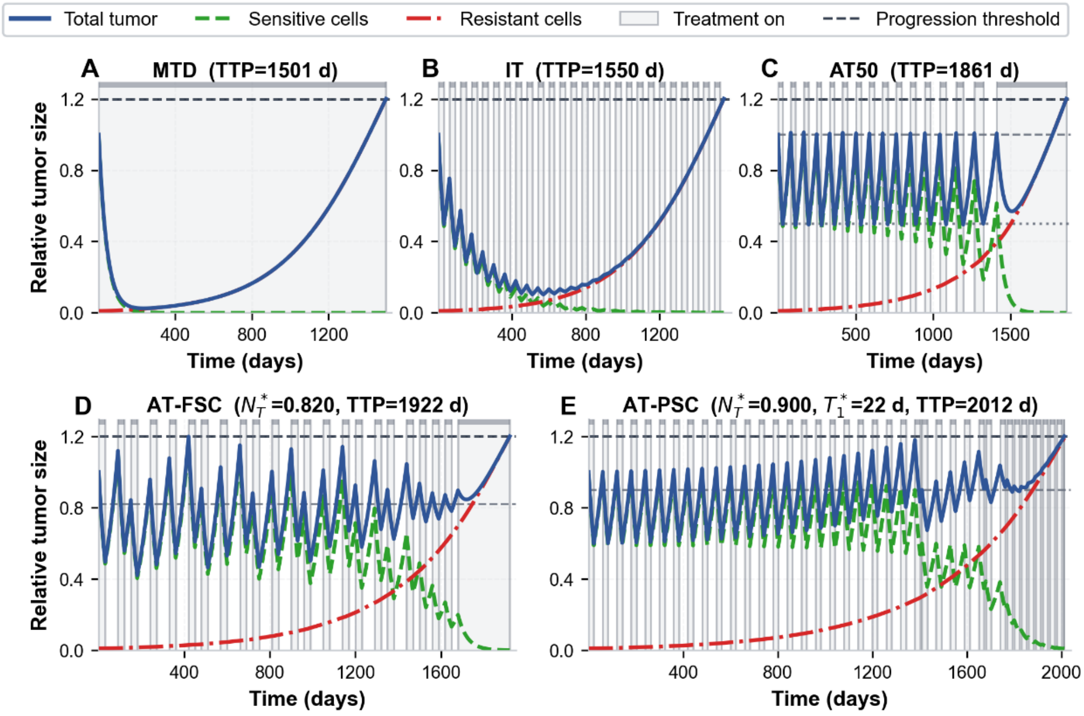

Fig E. Tumor-dynamics plots for Patient 20 under different treatment strategies.

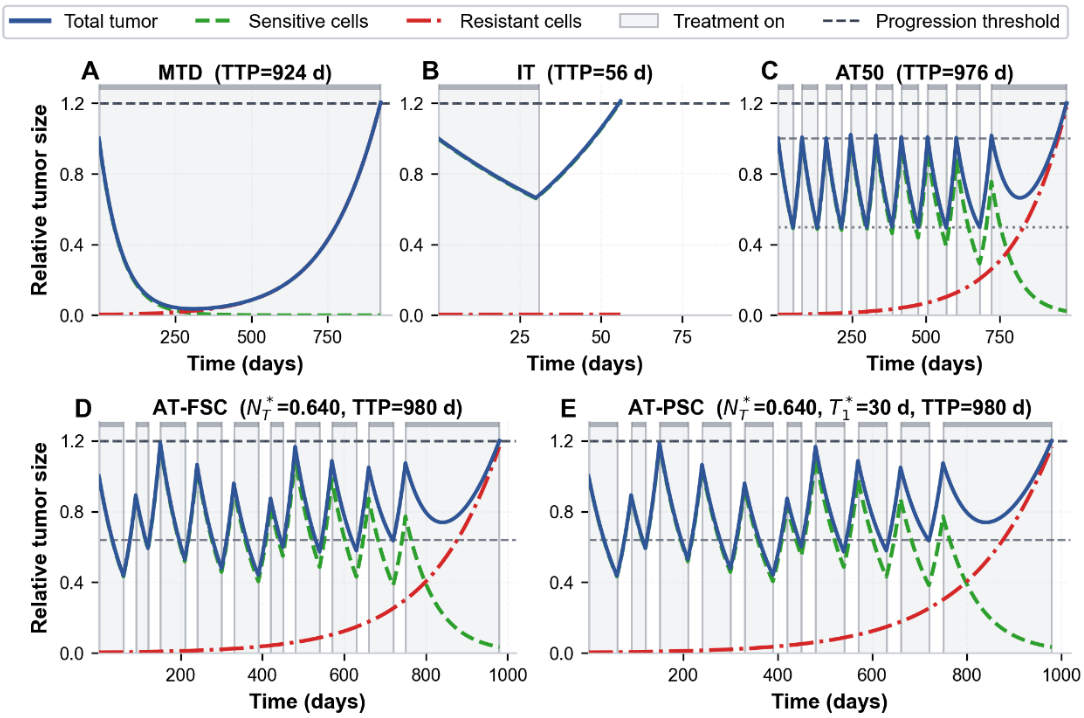

Fig F. Tumor-dynamics plots for Patient 12 under different treatment strategies.

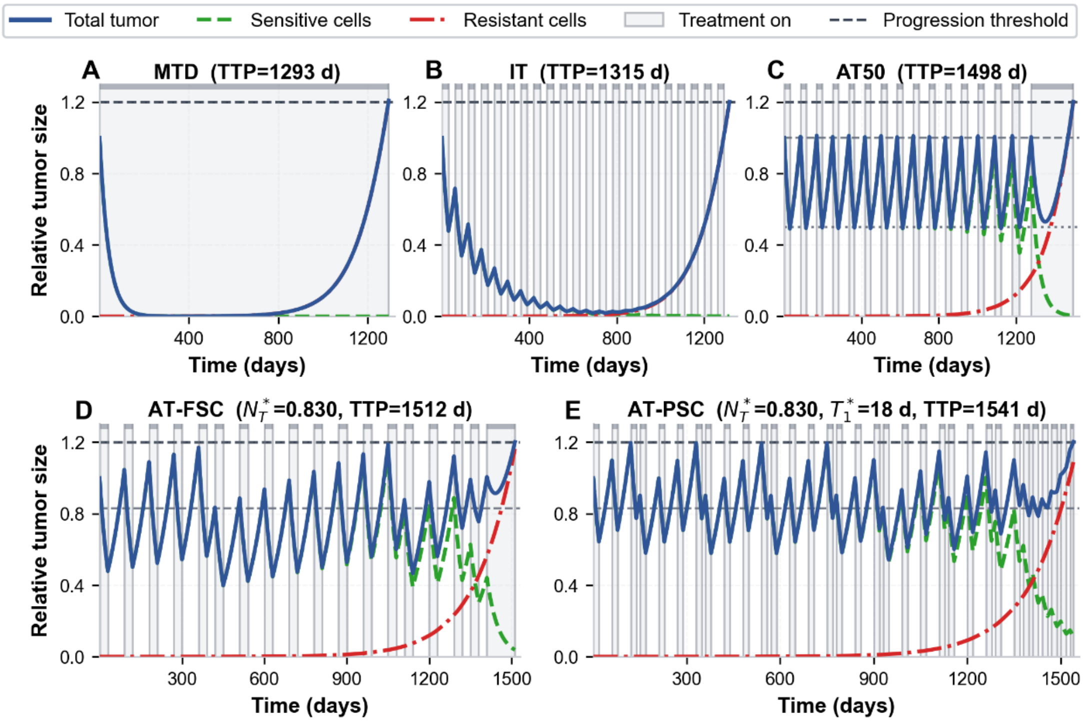

Fig G. Tumor-dynamics plots for Patient 99 under different treatment strategies.

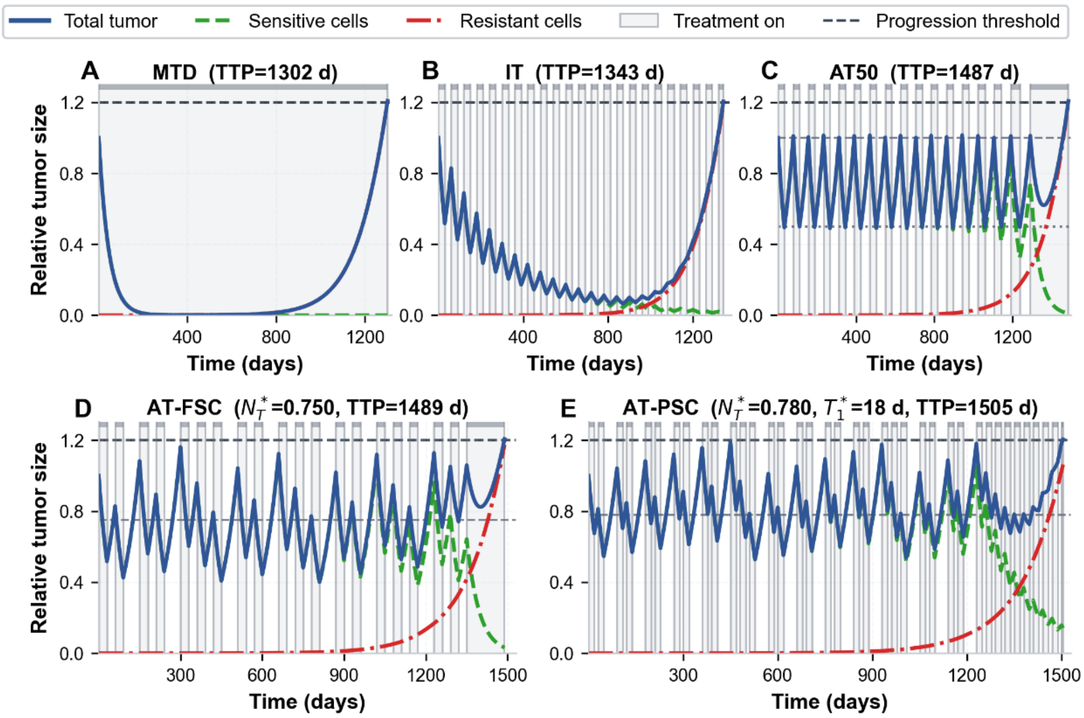

Fig H. Tumor-dynamics plots for Patient 101 under different treatment strategies.

Supplementary Note 3. Parameter-space analyses of AT-PSC performance across tumor growth profiles and treatment-window settings

Figs I–K provide additional parameter-space analyses supporting the robustness of the treatment-window effects shown in Fig. 4. Specifically, we present the remaining two-dimensional slices of the (r_S, r_R) parameter space by fixing either the sensitive-cell growth rate r_S or the resistant-cell growth rate r_R , and varying the other growth parameter together with the treatment window T_1. These heatmaps show that the advantage of AT-PSC over AT-FSC is not restricted to the two representative slices shown in Fig. 4A and Fig. 4B, but persists across a wider range of tumor growth profiles. We further provide fixed-treatment-window analyses and distribution plots to summarize how the improvement in TTP and the optimal treatment window vary across the explored parameter space. Together, these supplementary analyses demonstrate that AT-PSC retains therapeutic benefit across heterogeneous tumor growth dynamics, while also highlighting that the magnitude of benefit and the optimal treatment duration depend on the balance between sensitive- and resistant-cell growth rates.

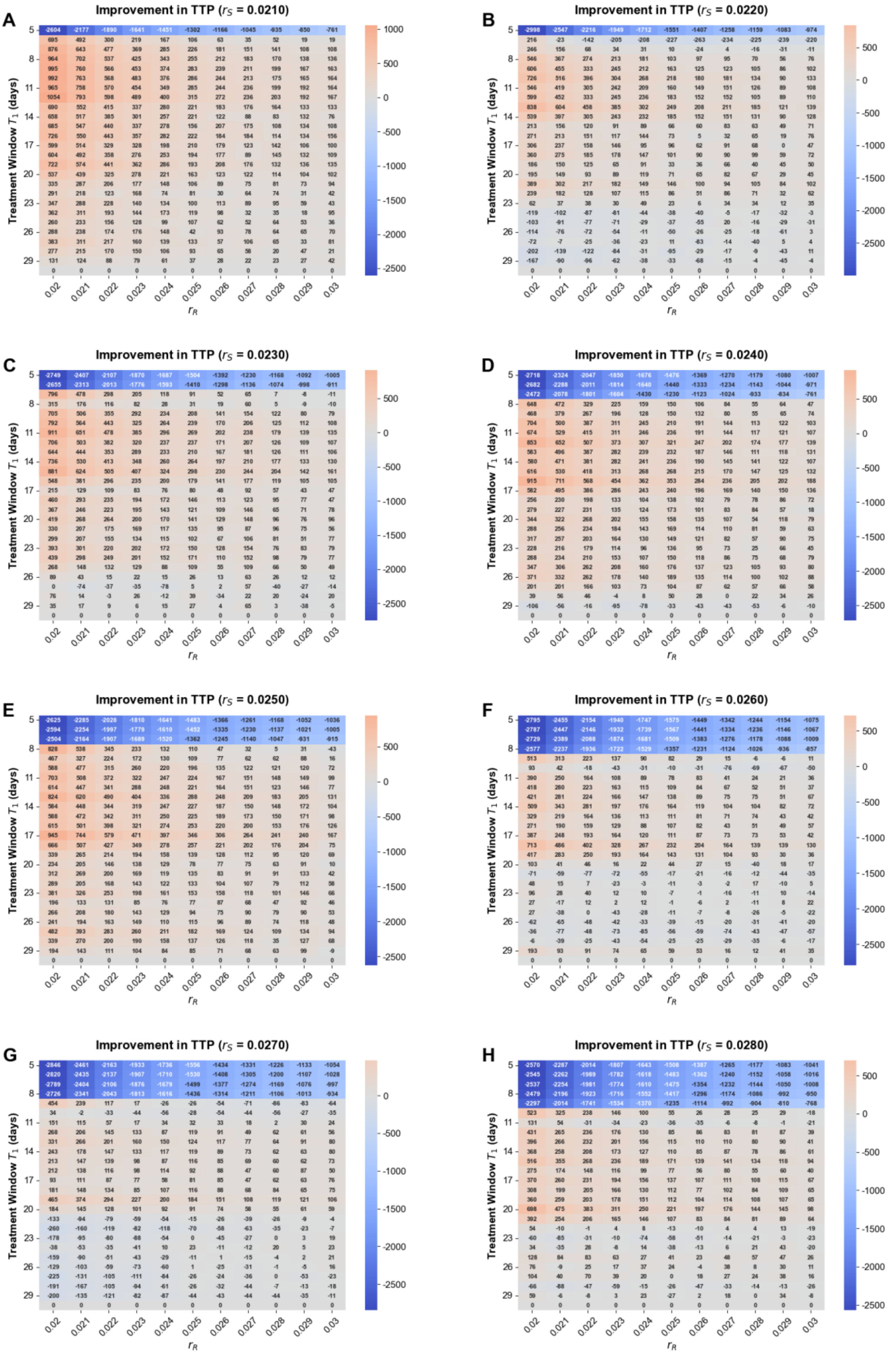

Fig I. Heatmaps of improvement in TTP for additional fixed-parameter scenarios (Part 1).

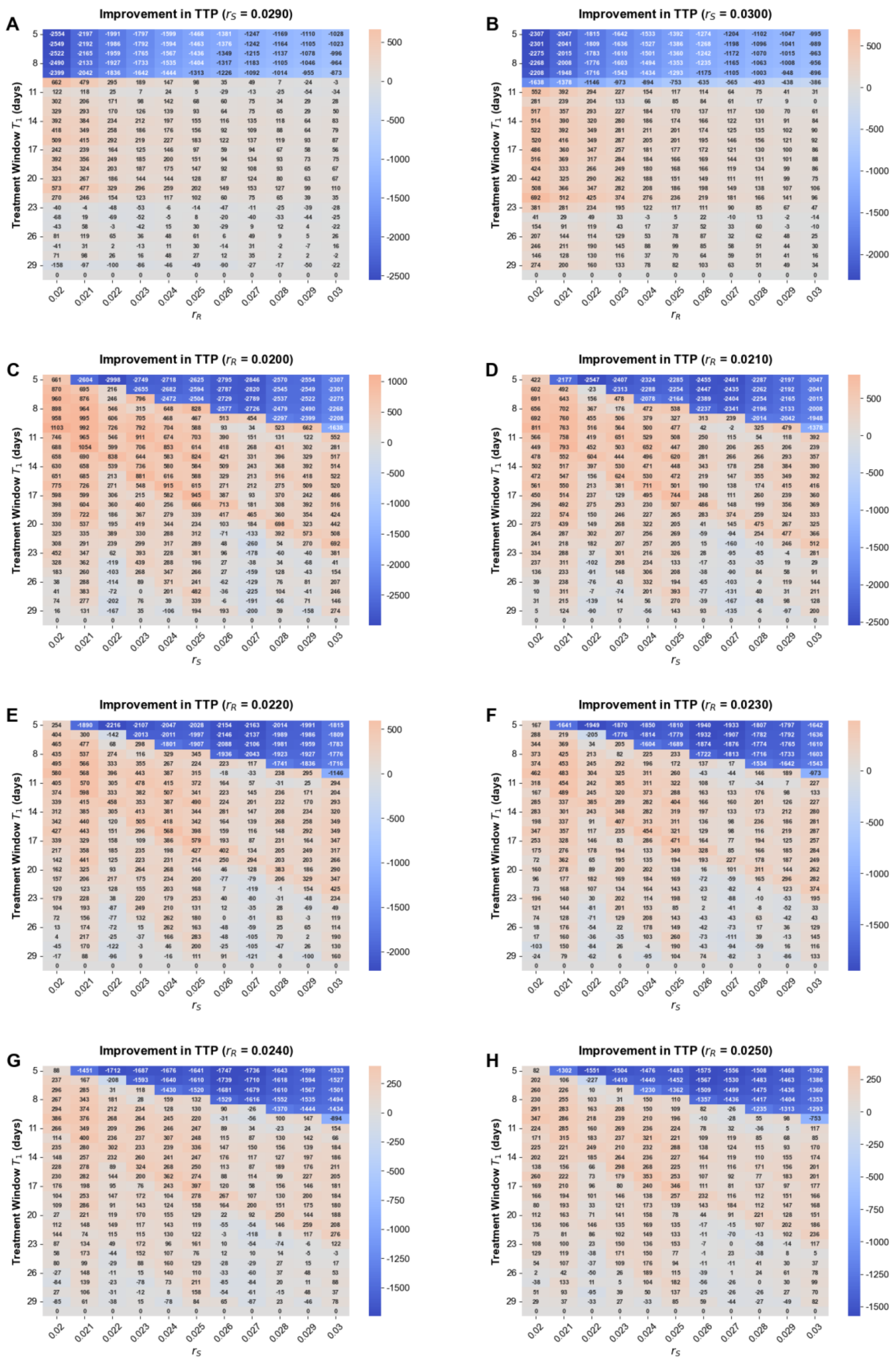

Fig J. Heatmaps of improvement in TTP for additional fixed-parameter scenarios (Part 2).

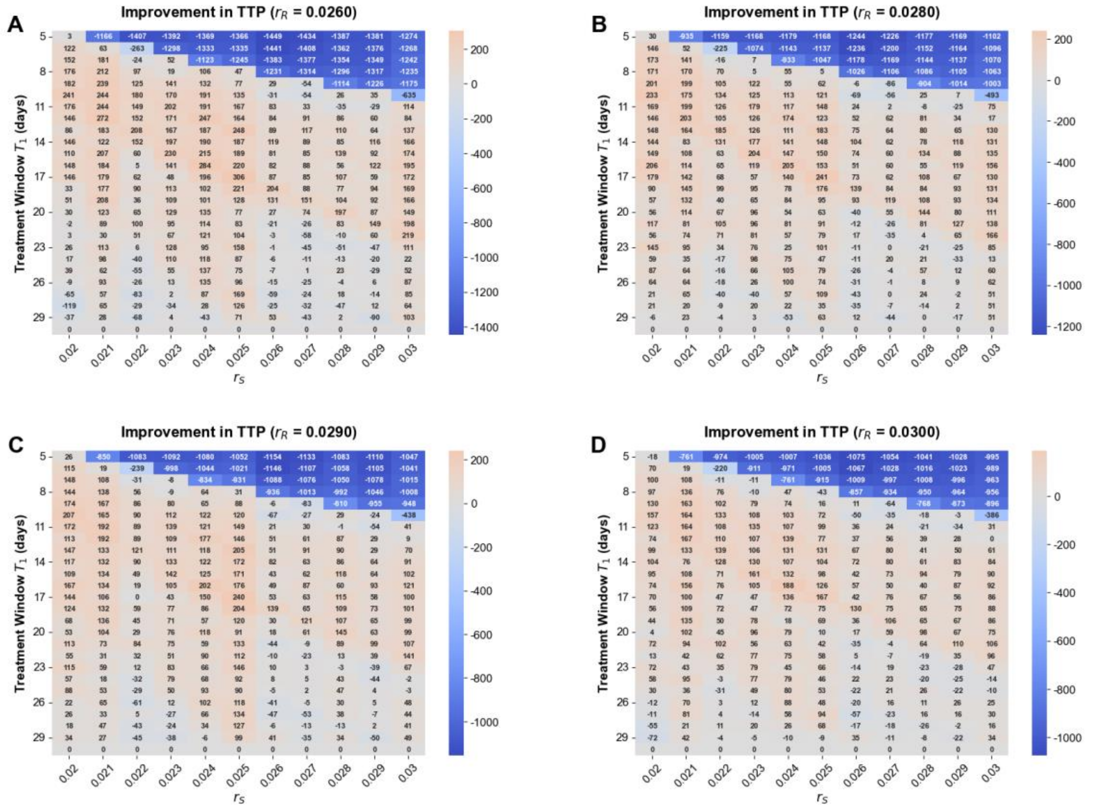

Fig K. Heatmaps of improvement in TTP for additional fixed-parameter scenarios (Part 3).

Supplementary Note 4. Empirically weighted analysis of treatment-window selection

To examine whether the apparent preference for a 14–30 schedule depends on uniform sampling of the explored (r_S, r_R) parameter space, we performed an empirically weighted analysis based on the fitted growth rates of the seven patients. Specifically, parameter combinations closer to the observed patient-specific (r_S, r_R) values were assigned larger weights, so that regions of parameter space more consistent with the fitted patient cohort contributed more strongly to the summary statistics. Fig L shows treatment-window improvement under empirical patient weighting and the corresponding parameter-space weighting map.

The results show that treatment windows of approximately 12–16 days substantially improve TTP relative to AT-FSC, consistent with the results shown in Fig. 4E. Therefore, the persistence of TTP improvement under this weighting scheme indicates that the preference for an intermediate treatment window is not merely a consequence of uniform parameter sampling. These results further support the "14–30" schedule as a feasible and clinically interpretable candidate schedule within the present modeling framework.

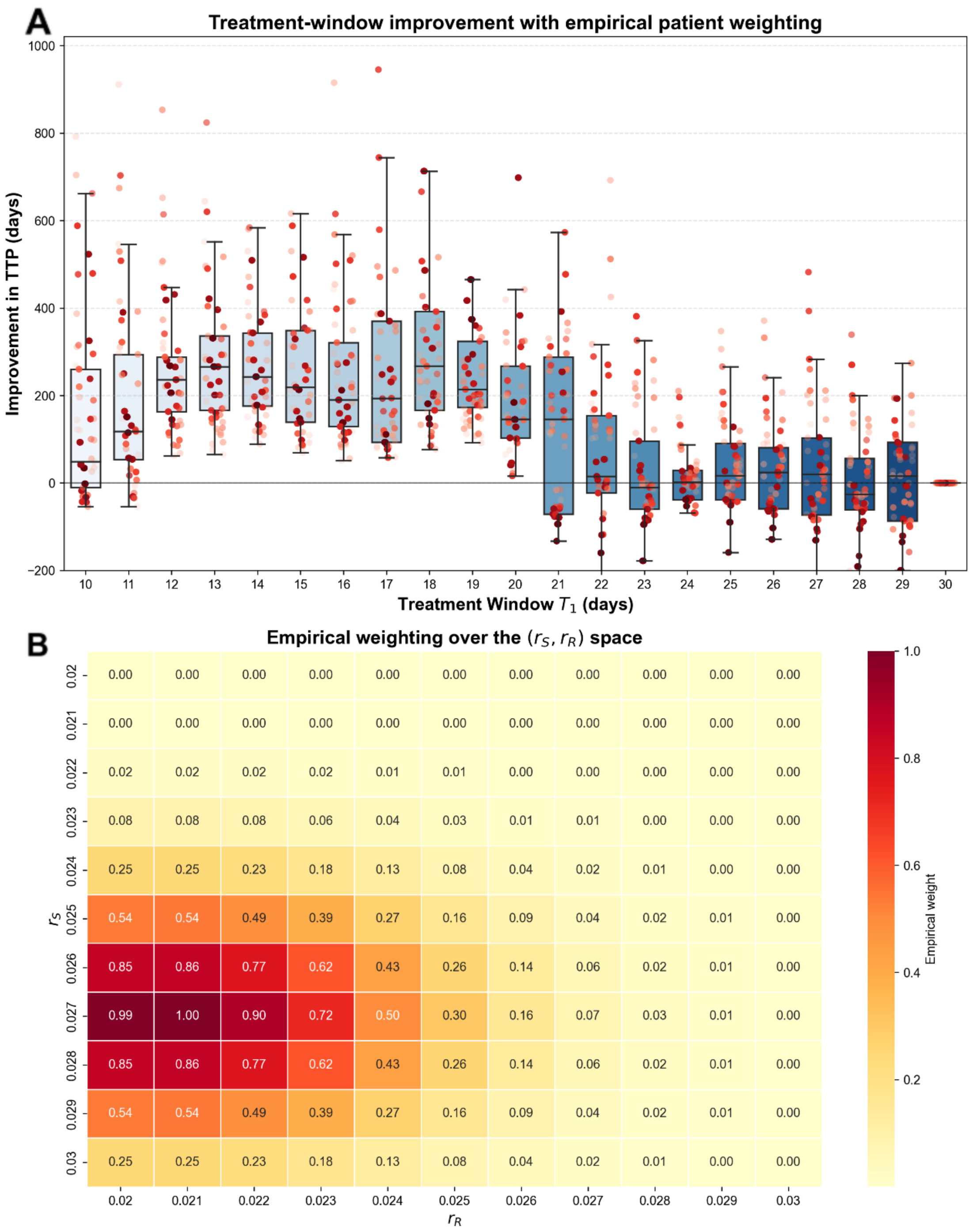

Fig L. A, Empirically weighted TTP improvement across fixed treatment windows. Box plots show the weighted distribution of TTP improvement for each treatment window T_1, based on resampling parameter combinations according to their proximity to the fitted patient-specific (r_S, r_R) values. Darker points indicate parameter combinations with larger empirical weights. **B,** Empirical weighting over the (r_S, r_R) space. Heatmap showing the relative weights assigned to parameter combinations according to their proximity to the fitted growth rates of the seven patients. Higher values indicate regions of parameter space that contribute more strongly to the empirically weighted summary statistics.

Supplementary Note 5. Robustness of AT-PSC to surveillance intervals and appointment delays

To complement the main-text results, we further present patient-level timing-robustness analyses for AT-PSC. Fig M shows the dependence of TTP on the surveillance interval for all seven patients. For each patient-specific parameter set, the AT-PSC schedule was re-optimized at each surveillance interval. In general, shorter surveillance intervals tend to yield longer TTP, although the magnitude of this effect differs substantially across patients. For comparison, Fig N presents the corresponding heatmaps of TTP for optimized AT-FSC and AT-PSC across selected surveillance intervals. The heatmaps further show patient-to-patient differences in how TTP changes with the surveillance interval. For both strategies, TTP generally decreases as the surveillance interval becomes longer, although the size of this change differs across patients. In addition, AT-PSC gives longer TTP than AT-FSC for most patients and surveillance intervals, suggesting that the benefit of partial-cycle treatment is maintained across different surveillance intervals.

Fig O compares the sensitivity of different adaptive therapy schedules to stochastic appointment delays for Patient 25 under a 30-day surveillance cycle. These delays were introduced to mimic missed appointments, holidays, and scheduling fluctuations in clinical monitoring. Specifically, appointment delays were sampled from an exponential distribution, with the mean delay increasing from 0 to 30 days. As shown in Fig O, in the absence of appointment delays, the optimized AT-PSC schedule achieved the longest TTP for Patient 25, indicating that this strategy can fully exploit the benefit of patient-specific optimization under ideal monitoring conditions. However, this optimized strategy was highly sensitive to appointment delays: even a small increase in the mean delay led to a rapid decrease in TTP. In contrast, moderately lowering the treatment threshold improved the stability of the strategy to some extent. For example, the AT-PSC schedule with fixed $N_T = 0.93$ maintained a relatively high TTP when the mean appointment delay was small, but its performance declined markedly as the delay further increased. When the threshold was further reduced to $N_T = 0.85$, the initial TTP of AT-PSC was lower than that of the optimized AT-PSC schedule, but its decline was more gradual over a wider range of delays. This suggests that a lower threshold allows treatment to be initiated earlier, thereby providing a buffer against missed or delayed monitoring visits and reducing the performance loss caused by missing critical monitoring time points. The AT-FSC schedules showed an overall similar trend. Taken together, these results suggest that, in clinically realistic settings with missed appointments and scheduling fluctuations, slightly lowering N_T or adopting a more conservative treatment-initiation rule may provide more stable and implementation-robust therapeutic performance.

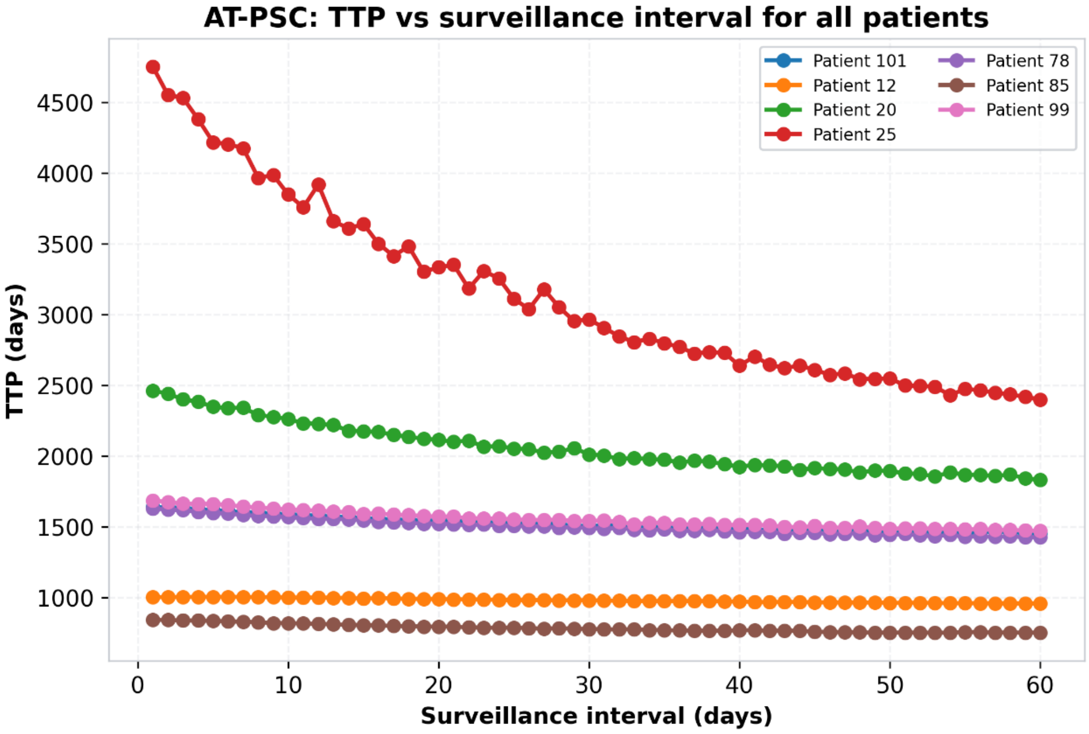

Fig M. Effect of surveillance interval on TTP under optimized AT-PSC across all patients.

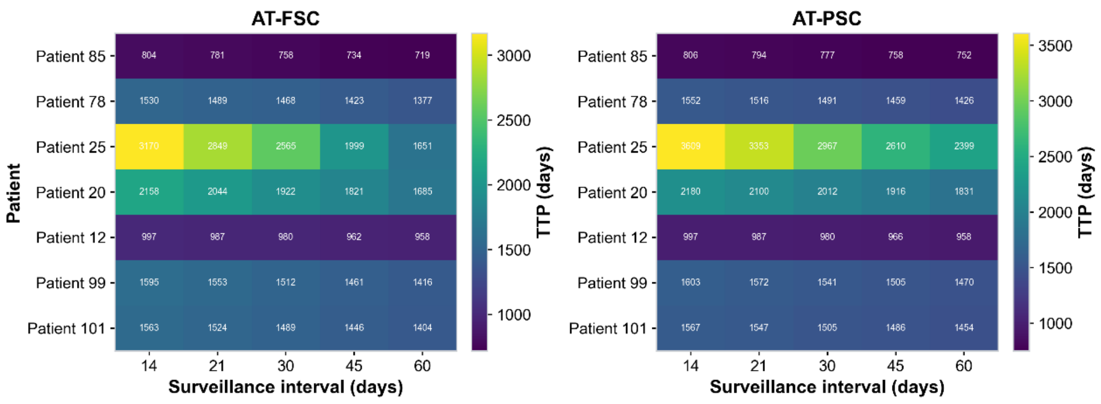

Fig N. TTP heatmaps for the optimized AT-FSC and AT-PSC strategies across patients and surveillance intervals.

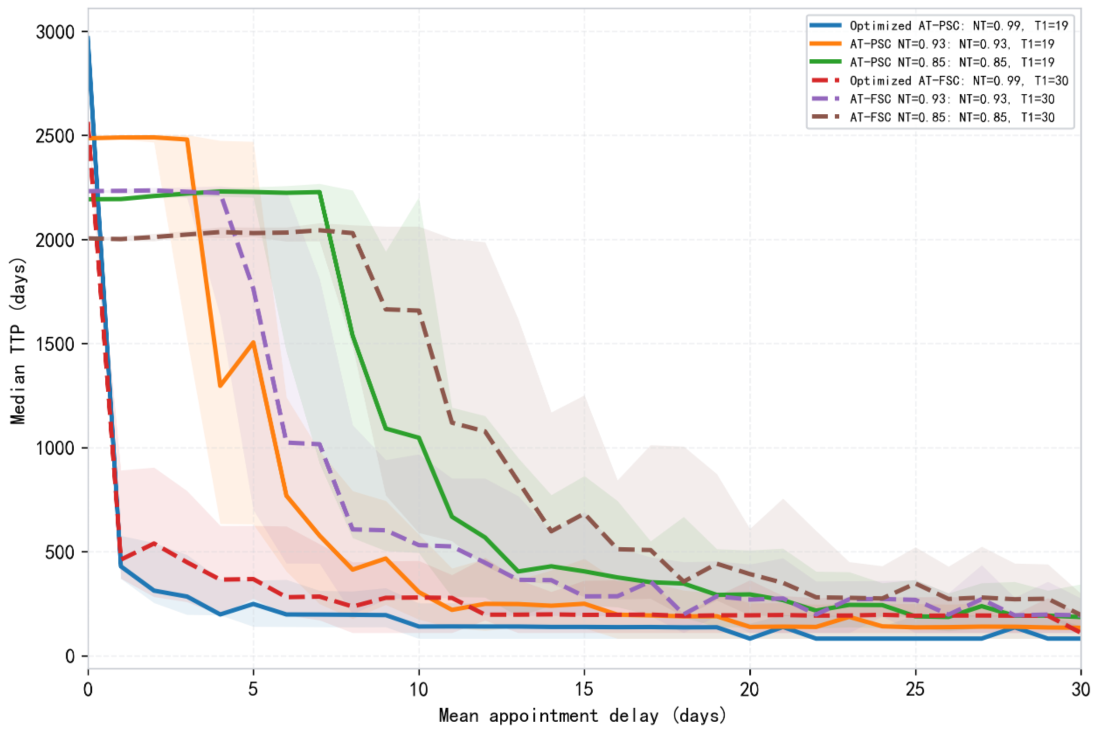

Fig O. Effect of missed-appointment-related delays on TTP for Patient 25. Solid lines represent AT-PSC schedules, whereas dashed lines represent AT-FSC schedules. The vertical axis shows the median TTP from 100 stochastic simulations, and the shaded regions indicate the interquartile range. The optimized AT-PSC schedule was obtained by grid search over N_T and T_1 under the no-delay setting, whereas the optimized AT-FSC schedule was obtained by grid search over N_T. The remaining curves correspond to AT-PSC or AT-FSC schedules with fixed thresholds $N_T = 0.93$ or $N_T = 0.85$.

Supplementary Note 6. Policy heatmaps across patients

Fig P visualizes the learned reinforcement-learning policy for each patient as a heatmap over the state space. For each patient (IDs 85, 78, 25, 20, 12, 99, and 101), we evaluate the trained policy network on a grid of relative tumor sizes (normalized tumor burden) and report the action probability distribution over treatment-window choices $T_1 \in \{0,1,\ldots,30\}$. Each panel shows $\pi_\theta(T_1 \mid s)$, where s is the relative tumor size; darker colors indicate higher probability of selecting a given T_1. To improve readability, probabilities below a small threshold are masked (shown as white), emphasizing the dominant decisions of the policy. These heatmaps provide an interpretable summary of how the RL controller adapts the recommended treatment window T_1 as tumor burden changes, and how this mapping differs across patient-specific parameterizations.

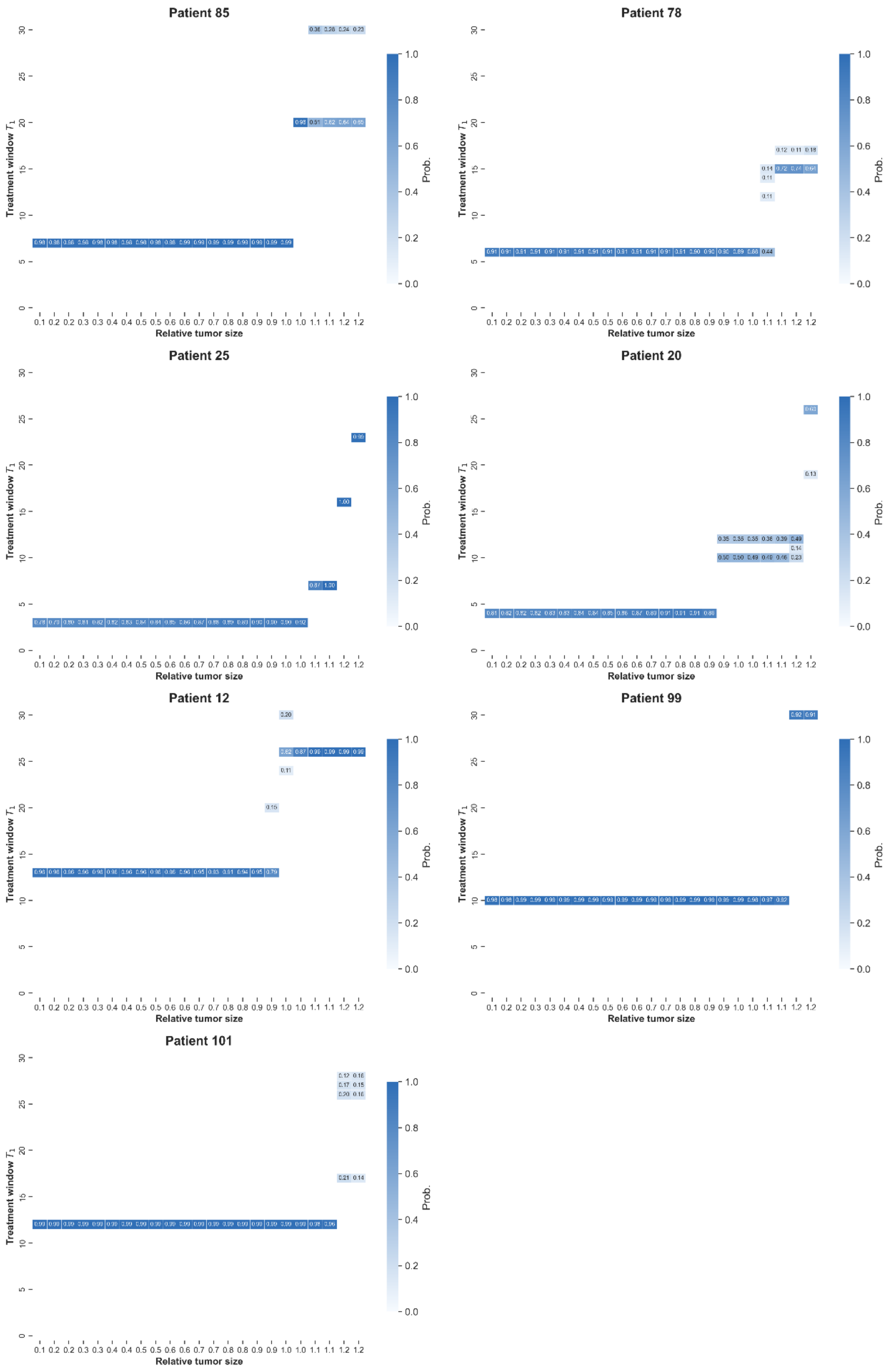

Fig P. Heatmap visualization of $\pi_\theta(T_1 \mid s)$ across patients

Supplementary Note 7. Virtual patient parameters

The parameters used in the Lotka-Volterra model are taken from clinical data (26,45) and outlined in Table A.

Table A. Patient-specific parameter values (27) for the Lotka-Volterra model

Patient ID	S_0	R_0	d_D	d_R	d_S	K	r_R	r_S
85	0.09594	0.00406	1.5	0.00877	0.00877	1	0.01436	0.027
78	0.1	1.00×10^{-6}	1.5	0.01242	0.01242	1	0.02209	0.027
25	0.42567	4.26×10^{-6}	1.5	0.00787	0.00787	1	0.02079	0.027
20	0.09902	0.00098	1.5	0.01119	0.01119	1	0.01487	0.027
12	0.09966	0.00034	1.5	0.00133	0.00133	1	0.00791	0.027
99	0.1	1.25×10^{-6}	1.5	0.012	0.012	1	0.0212	0.027
101	0.10275	1.03×10^{-6}	1.5	0.00949	0.00949	1	0.01876	0.027

Supplementary Note 8. Protocol-specific optimal thresholds for the threshold-guided adaptive therapy strategies under the Patient 25 parameter set.

For each protocol, the optimal threshold N_T^* is defined as the threshold value that maximizes the corresponding TTP curve.

Table B. Optimal thresholds and corresponding TTP values for each protocol under the Patient 25 parameter set.

**Protocol**	**Estimated N_T^***	**TTP**
AT50($N_T, 0.5N_0$)	1.16	2100
AT-FSC($N_T, 30; 30$)	0.99	2565
AT-PSC($N_T, 15; 30$)	0.98	2755

Supplementary Note 9. Optimization cost of rule-based treatment strategies

To clarify the relative optimization effort across the rule-based treatment strategies, we summarize in Table C the tunable parameters, candidate settings, optimization cost per patient, and mean runtime per patient for MTD, IT, AT50, AT-FSC, and AT-PSC. As expected, AT-PSC requires a larger optimization effort than AT-FSC because it is optimized over both the tumor-size threshold N_T and the treatment window T_1, whereas AT-FSC is optimized only over N_T. MTD, IT, and AT50 do not require grid-based parameter optimization in the present implementation and therefore incur only a single forward simulation per patient.

Table C. Optimization settings and computational cost of the rule-based treatment strategies.

**Strategy**	**Tunable parameters**	**Candidate settings**	**Optimization cost per patient**	**Mean time per patient(s)**
MTD	0	1	1 forward simulation	0.033895
IT	0	1	1 forward simulation	0.031280
AT50	0	1	1 forward simulation	0.051530
AT-FSC	1(N_T)	101	101 search simulations + 1 final simulation	3.170121
AT-PSC	2(N_T, T_1)	3030	3030 search simulations + 1 final simulation	68.075448

Supplementary Note 10. Outcomes under adaptive RL(0,1,…,30) compared with other treatment strategies

Table D summarizes the outcomes for different patients under various treatment strategies. For each patient, we report the TTP (days) and total treatment dose (%) for RL(0,1,…,30), AT-PSC, AT-FSC, and AT50. For RL(0,1,…,30), the TTP is presented as the median together with the 95 % quantile range (QR) obtained from stochastic evaluations. Dose percentages for AT-PSC, AT-FSC, and AT50 are reported alongside the relative difference compared with the RL dose baseline.

Table D. Comparison of TTP and dose across different treatment strategies.

ID	TTP（Days）				Dose(%)			
	RL[1]	RL (95% QR) [1]	AT-FSC	AT-PSC	RL[1]	AT-PSC[2]	AT-FSC[2]	AT50[2]
85	778	[748, 790]	758	777	51.67	56.94%(+10.20%)	60.47%(+17.03%)	65.56%(+26.88%)
78	1494	[1394, 1516]	1468	1491	35.28	36.26%(+2.78%)	38.73%(+9.78%)	40.76%(+15.53%)
25	3147	[3014, 3179]	2565	2967	34.02	35.24%(+3.59%)	39.20%(+15.23%)	48.83%(+43.53%)
20	2076	[2039, 2100]	1922	2012	40.02	42.77%(+6.87%)	46.96%(+17.34%)	52.85%(+32.06%)
12	984	[964, 986]	980	980	71.77	72.48%(+0.99%)	72.48%(+0.99%)	73.49%(+2.40%)
99	1545	[1467, 1576]	1512	1541	36.81	38.13%(+3.59%)	40.52%(+10.08%)	44.70%(+21.43%)
101	1499	[1375, 1545]	1489	1505	42.25	44.62%(+5.61%)	47.65%(+12.78%)	49.60%(+17.40%)

[1]RL denotes the RL(0,1,…,30) strategy.

[2]Values in parentheses indicate the percentage difference relative to the RL dose baseline, calculated as (Strategy Dose－RL Dose) / RL Dose ×100%.

Supplementary Note 11. Computational cost of the RL-based strategies

To clarify the computational expense of the RL-based strategies under different action-space designs, we summarize in Table E the training configuration and nominal computational budget of the two PPO implementations used in this study. Both implementations share the same environment horizon, PPO optimization scheme, and network backbone, and differ primarily in the size of the action space. In the constrained-action setting, the agent chooses between two cycle-level actions corresponding to no treatment or a fixed 19-day treatment window. In the broader-action setting, the agent directly selects the treatment duration from 0 to 30 days for each cycle. Since each episode contains at most 365 decision steps and each decision step simulates up to 30 daily tumor-dynamics updates, the nominal upper bound is 10,950 daily ODE updates per episode.

Table E. Training configurations and computational cost of the RL-based strategies under different action-space designs.

RL Strategy	RL(0, 19)	RL(0,1,…,30)
**Action space**	Discrete (2)	Discrete (31)
**Action meaning**	0 = no treatment; 1 = treat 19 days	choose treatment window from 0 to 30 days
**Policy output dimension**	2	31
**Trainable parameters**	45019	45338
**Training episodes**	100000	100000
**Max decision steps per episode**	365	365
**Mean runtime per training run (s)**	7786.71	11054.39
